# A regulated NMD mouse model supports NMD inhibition as a viable therapeutic option to treat genetic diseases

**DOI:** 10.1242/dmm.044891

**Published:** 2020-08-27

**Authors:** Josh Echols, Amna Siddiqui, Yanying Dai, Viktoria Havasi, Richard Sun, Aneta Kaczmarczyk, Kim M. Keeling

**Affiliations:** Department of Biochemistry and Molecular Genetics, University of Alabama at Birmingham, Birmingham, AL 35294, USA

**Keywords:** Nonsense-mediated mRNA decay, NMD, UPF1, Dominant negative, Mouse, Inhibition, Characterization

## Abstract

Nonsense-mediated mRNA decay (NMD) targets mRNAs that contain a premature termination codon (PTC) for degradation, preventing their translation. By altering the expression of PTC-containing mRNAs, NMD modulates the inheritance pattern and severity of genetic diseases. NMD also limits the efficiency of suppressing translation termination at PTCs, an emerging therapeutic approach to treat genetic diseases caused by in-frame PTCs (nonsense mutations). Inhibiting NMD may help rescue partial levels of protein expression. However, it is unclear whether long-term, global NMD attenuation is safe. We hypothesize that a degree of NMD inhibition can be safely tolerated after completion of prenatal development. To test this hypothesis, we generated a novel transgenic mouse that expresses an inducible, dominant-negative form of human *UPF1* (*dnUPF1*) to inhibit NMD in mouse tissues by different degrees, allowing us to examine the effects of global NMD inhibition *in vivo*. A thorough characterization of these mice indicated that expressing *dnUPF1* at levels that promote relatively moderate to strong NMD inhibition in most tissues for a 1-month period produced modest immunological and bone alterations. In contrast, 1 month of *dnUPF1* expression to promote more modest NMD inhibition in most tissues did not produce any discernable defects, indicating that moderate global NMD attenuation is generally well tolerated in non-neurological somatic tissues. Importantly, a modest level of NMD inhibition that produced no overt abnormalities was able to significantly enhance *in vivo* PTC suppression. These results suggest that safe levels of NMD attenuation are likely achievable, and this can help rescue protein deficiencies resulting from PTCs.

## Abstract (precis)

**Summary:** Nonsense-mediated mRNA decay can be inhibited after completion of mammalian prenatal development without adverse effects in non-neurological, somatic tissues, indicating that such inhibition might be a viable therapeutic strategy.

## INTRODUCTION

A third of all gene lesions that are associated with human genetic diseases generate a premature termination codon (PTC) in the open reading frame (ORF) of an mRNA (Khajavi et al., 2006). A PTC ends translation of an mRNA before a full-length protein is generated. In addition, a PTC often triggers nonsense-mediated mRNA decay (NMD), a conserved eukaryotic mRNA surveillance pathway that degrades PTC-containing mRNAs to prevent their translation. By mediating the expression of PTC-containing transcripts, NMD efficiency alters the inheritance pattern and the clinical severity of genetic diseases in patients harboring PTC-forming mutations (Bhuvanagiri et al., 2010; Khajavi et al., 2006). In addition, by reducing the pool of PTC-containing mRNAs available for translation, NMD efficiency also affects the efficacy of so-called ‘readthrough’ approaches that suppress translation termination at in-frame PTCs to restore partial, but physiologically significant, levels of full-length, functional protein (Khajavi et al., 2006; Linde et al., 2007b; Kerem et al., 2008; Wilschanski et al., 2003). Taken together, these data suggest that attenuating NMD represents a potential therapeutic approach to help rescue deficient protein in patients who harbor a PTC.

Evidence indicates that inhibiting NMD to increase the pool of PTC-containing mRNAs available for translation and subsequent readthrough can help restore expression of functional protein. For example, inhibiting NMD by knocking down NMD factor expression in cultured epithelial cells from cystic fibrosis patients increases the abundance of PTC-containing *CFTR* mRNA and restores greater CFTR protein function than with readthrough approaches alone (Linde et al., 2007b). Furthermore, short-term attenuation of NMD *in vivo* using the inhibitor compound, NMDI-1, (Durand et al., 2007) enhances PTC suppression in a mouse model of the lysosomal storage disease, mucopolysaccharidosis I-Hurler (MPS I-H) (Keeling et al., 2013). These data suggest that inhibiting NMD can significantly enhance the effectiveness of readthrough therapies.

Restoring the expression of truncated proteins by inhibiting NMD alone can also help alleviate some disease phenotypes. For example, restoring truncated collagen VI expression in Ullrich's disease patient fibroblasts using the phosphoinositide 3-kinase inhibitors wortmannin and caffeine, which inhibit the NMD factor SMG1, partially rescues extracellular matrix function *in vitro* (Usuki et al., 2004). These data suggest that inhibiting NMD may be a way to restore expression of functional truncated proteins for some genetic diseases.

Although these previous studies indicate that short-term NMD inhibition can help rescue partial expression of proteins that are deficient due to a PTC, it has remained unclear whether inhibiting NMD therapeutically is feasible due to potential safety issues. Apprehension over the safety of attenuating NMD stems from several lines of evidence. First, NMD not only regulates PTC-containing mRNAs resulting from genomic mutations, it also controls the expression of ∼10% of the mammalian transcriptome (Mendell et al., 2004). Physiological NMD substrates include transcripts containing upstream ORFs (uORFs) in the 5′ untranslated region (UTR), transcripts with long or intron-containing 3′ UTRs, transcripts containing selenocysteine codons and products of alternative splicing that harbor PTCs. Some of these NMD substrates are known to participate in diverse cellular pathways such as DNA synthesis, cell-cycle progression, telomere length homeostasis and cellular stress responses (Isken and Maquat, 2008), suggesting that NMD inhibition could alter cellular functions. Second, null mutations of many NMD factors result in embryonic lethality in mice, suggesting that NMD is essential for normal mammalian development (Medghalchi et al., 2001; Weischenfeldt et al., 2008; McIlwain et al., 2010). Third, copy number variants of certain NMD factors have been shown to be associated with multiple forms of intellectual disability in patients (Tarpey et al., 2007; Nguyen et al., 2013). These data suggest that strong NMD inhibition during development can have severe adverse effects. However, several studies have also suggested that some degree of NMD perturbation is well tolerated since NMD efficiency has been found to vary by 2- to 4-fold among the general population (Linde et al., 2007a; Viegas et al., 2007; Seoighe and Gehring, 2010; Nguyen et al., 2014).

We hypothesize that a level of global NMD inhibition can be achieved after the completion of prenatal development without the formation of severe, aberrant phenotypes. To test our hypothesis, we generated transgenic mice that express a dominant-negative form of the human *UPF1* cDNA (*dnUPF1*) under the control of an inducible tetracycline response element. This allowed dnUPF1 protein expression, and the subsequent degree of NMD inhibition, to be tightly and reproducibly controlled during and after development by doxycycline dosing. This new inducible *dnUPF1* transgenic mouse enabled us to examine the *in vivo* effects of global NMD attenuation on gene expression, morphology and physiology.

We found that expressing *dnUPF1* at levels that moderately to strongly inhibited NMD in many mouse tissues for a 1-month period modestly altered the immunological profile in transgenic mice. In female transgenic mice, we also observed that this degree of NMD inhibition led to minor changes in bone morphology. In contrast, expressing *dnUPF1* at levels that mildly to moderately inhibited NMD in most mouse tissues for a 1-month period did not lead to any discernable changes in the biochemistry, morphology or physiology of somatic tissues from *dnUPF1* transgenic mice. In addition, we found that a mild-to-moderate level of *in vivo* NMD inhibition was able to significantly increase the abundance of a PTC-containing mRNA generated by a genomic nonsense mutation, enhancing the amount of protein function restored by readthrough.

Interestingly, we observed a significant change in NMD substrate abundance in neurological tissues with very modest *dnUPF1* expression, suggesting that neurological tissues might be particularly responsive to changes in NMD efficiency. Further evaluation of neurological tissues will be required to determine whether abnormalities form upon long-term NMD inhibition. However, we found no abnormalities in non-neurological somatic tissues upon moderate NMD attenuation. Taken together, our results suggest that mild-to-moderate NMD attenuation appears to be well-tolerated in most somatic tissues and could potentially be beneficial as a therapeutic approach to treat genetic diseases resulting from PTCs.

## RESULTS

### Generation of *dnUPF1* transgenic mice

UPF1, a phosphoprotein and ATP-dependent helicase, is a highly conserved factor that is essential for all known branches/forms of NMD (Nicholson et al., 2014). To examine the *in vivo* effects of globally inhibiting NMD, we generated transgenic mice that express a human *UPF1* cDNA carrying an R844C mutation. Introduction of this mutation results in the production of a stable, mutant UPF1 protein that can assemble into NMD complexes but lacks helicase activity, which is necessary for NMD. Thus, this mutant UPF1 acts in a dominant-negative manner by reducing the number of functional NMD complexes (Sun et al., 1998; Frischmeyer-Guerrerio et al., 2011). Henceforth, we refer to the *UPF1-R844C* allele as *dnUPF1*.

**Fig. 1.**
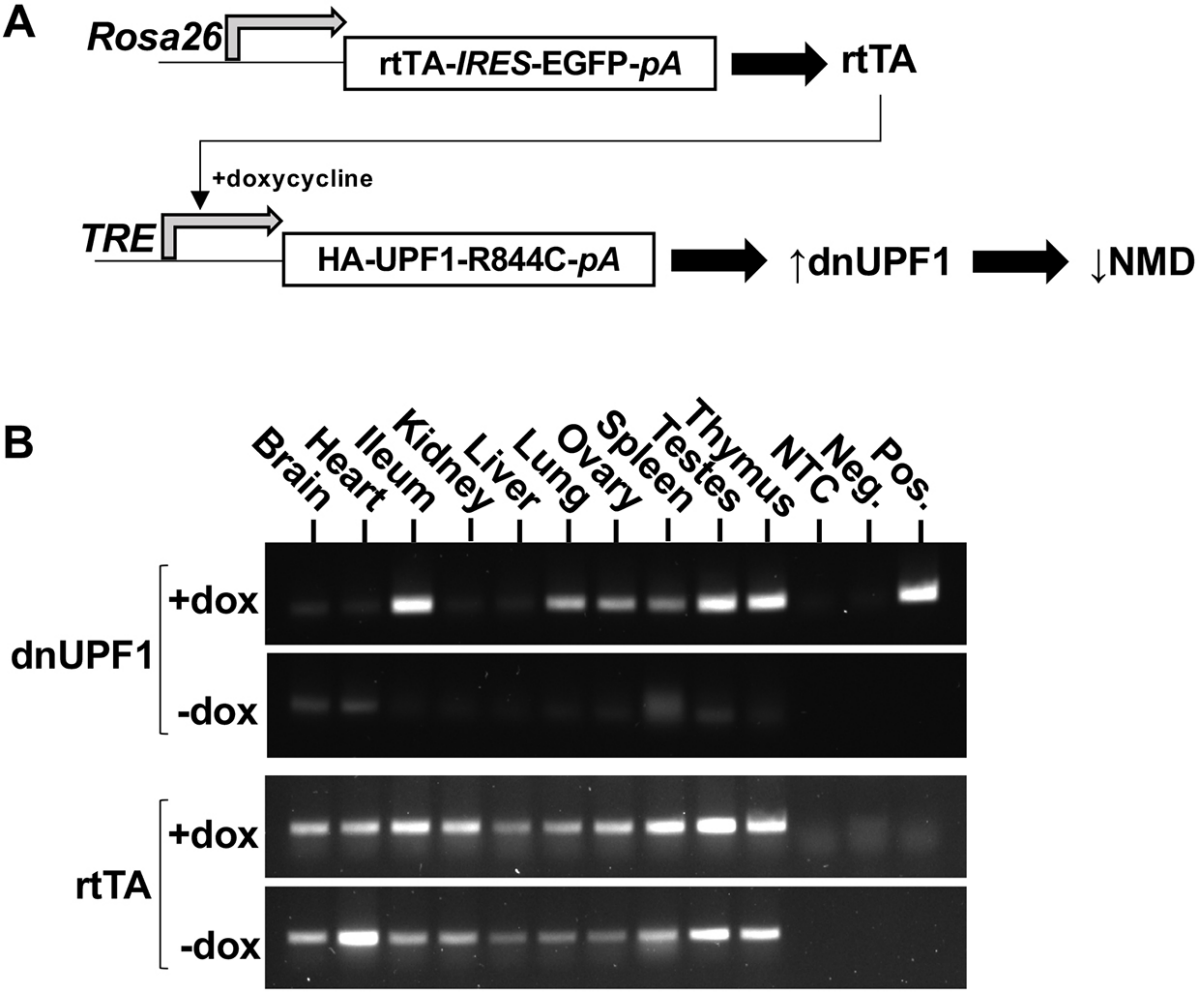
**Generation of transgenic mice that express a dominant-negative *UPF1* (*dnUPF1*) transgene via a TET-ON system.** (A) Schematic of the TET-ON expression system. A reverse tetracycline transactivator (rtTA) (expressed from the endogenous mouse *Rosa26* locus) elicits HA-epitope tagged dnUPF1 expression (via the tetracycline response element, *TRE*) in the presence of doxycycline (dox). (B) RT-PCR was performed to examine the extent of *dnUPF1* mRNA expression in different tissues when mice were treated with 500 mg/ml dox for 7 days. NTC, non-template control; Neg., no transgene control; Pos., *dnUPF1*-expressing plasmid control. *rtTA* serves as a PCR control. A representative gel is shown from four similar, independent experiments.

*dnUPF1* transgene expression was regulated using a TET-ON system (Fig. 1A). The *dnUPF1* cDNA was placed under the control of a tetracycline response element (*TRE*), consisting of seven direct repeats of a 42-bp sequence containing the *tet* operator located just upstream of the minimal *CMV* promoter. This promoter has been shown to function in a wide range of tissues, including embryonic tissues (Sun et al., 2007). The *SV40* transcriptional terminator (*pA*) was also present in the construct. A reverse tetracycline transactivator (*rtTA*) constitutively expressed from the *Rosa26* locus was used to activate *dnUPF1* expression from the *TRE* in the presence of doxycycline (dox), a tetracycline derivative. Because mouse and human UPF1 share 98% identity at the amino acid level, a hemagglutinin (HA) epitope tag was fused to the N terminus of the *dnUPF1* cDNA to distinguish *dnUPF1* expression from endogenous mouse *Upf1* expression. We generated two transgenic lines, one that carried three to four copies of the *dnUPF1* cDNA, and another that carried between 12 and 14 copies of the *dnUPF1* cDNA, according to digital PCR. We chose to perform our characterization using the line carrying the highest number of transgene copies to maximize *dnUPF1* expression. This line also showed the broadest tissue expression pattern. To simplify breeding and maximize *dnUPF1* expression, all mice were bred to be homozygous for both the *rtTA* and *dnUPF1*.

To identify the tissues in which the *dnUPF1* transgene was expressed, 12-week-old *dnUPF1* mice were administered 500 mg/ml dox in a sugar-free Kool-Aid^®^ vehicle for 7 days. Total RNA was harvested from multiple tissues of dox-treated and control mice, reverse transcribed (RT) to cDNA, and then subjected to PCR using primers that anneal specifically to *dnUPF1* (Fig. 1B). We found that *dnUPF1* mRNA was expressed in many tissues when *dnUPF1* mice were administered dox, with the most robust expression observed in the ileum, lung, ovary, spleen, testes and thymus. In *dnUPF1* mice administered vehicle only (−dox), little to no *dnUPF1* mRNA expression was observed, indicating the precise, tight control of the TET-ON expression system. The variability of *dnUPF1* transgene expression among various tissues might potentially be explained by tissues having differential: (1) transcriptional activity from the chromosomal loci into which the transgene became integrated, (2) promoter activity, (3) *rtTA* expression, or (4) doxycycline penetration.

**Fig. 2.**
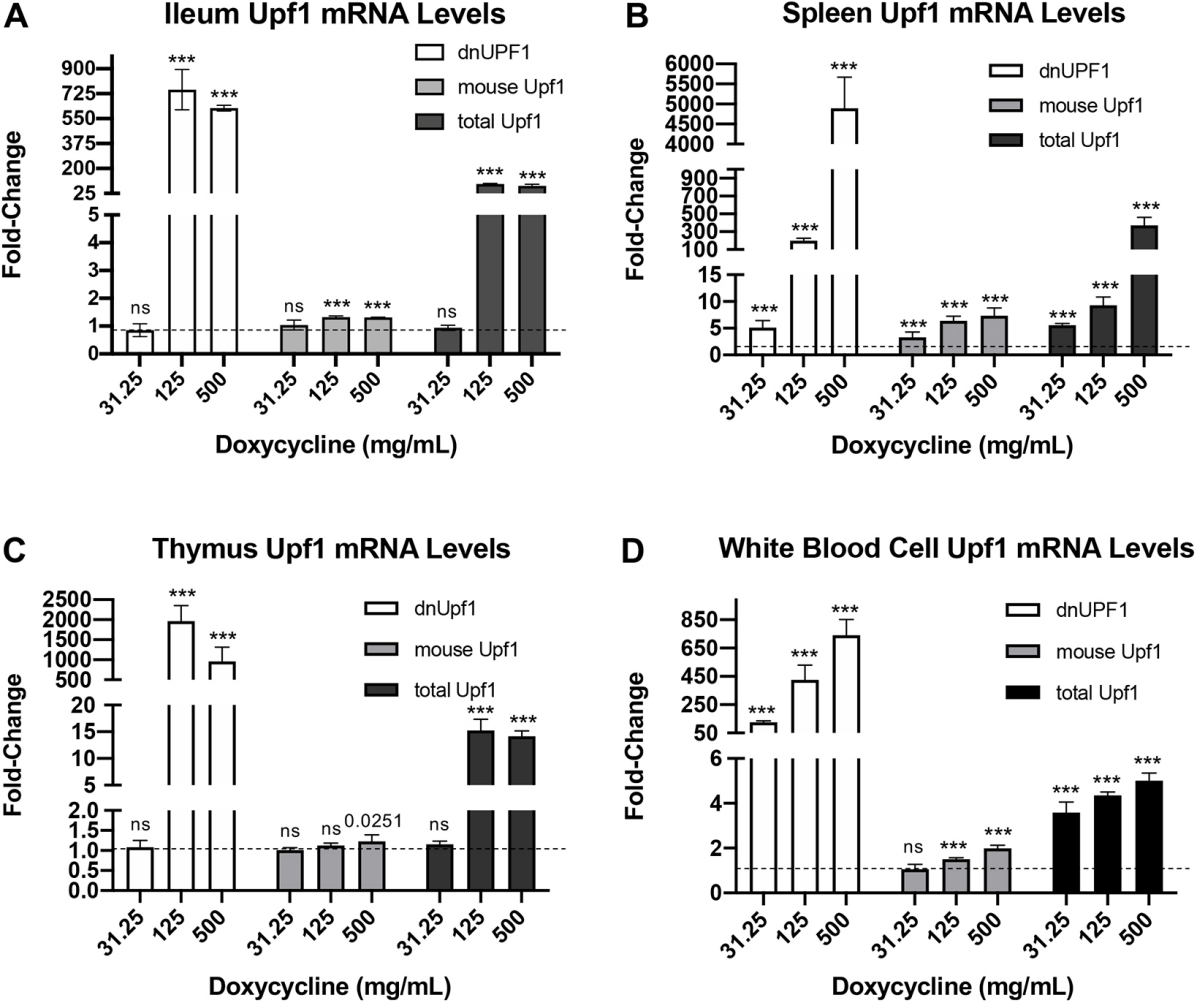
***dnUPF1*** **is expressed in response to doxycycline dosing within multiple transgenic mouse tissues.** qPCR was performed to examine the levels of *dnUPF1* mRNA, endogenous mouse *Upf1* mRNA, and total *UPF1* mRNA (*dnUPF1*+mouse *Upf1*) relative to *Gapdh* in (A) ileum, (B) spleen, (C) thymus, and (D) circulating white blood cells. Mice were treated with doxycycline (dox) for 28 days prior to analysis. The data are expressed as the mean fold change (±s.d.) in mRNA levels in dox-treated mice relative to vehicle-only controls (indicated by dashed lines). *n*=6, from three mice per cohort. An unpaired, two-tailed *t*-test was used to determine exact *P*-values when comparing dox-treated mice to vehicle-only controls. ***P<0.0001; ns, *P*>0.05.

We next quantified *dnUPF1* mRNA expression in mouse tissues. Three-week-old mice were treated with a range of dox doses for 28 days prior to tissue collection. After RNA was isolated from tissue lysates and reverse transcribed into cDNA, qPCR was performed, with *Gapdh* serving as a normalization control. qPCR results indicated a robust increase in *dnUPF1* expression in dox-treated mice compared to that in vehicle controls for all tissues examined (Fig. 2A-D), with the greatest elevations of *dnUPF1* found in the spleen (Fig. 2B) and thymus (Fig. 2C). Notably, *dnUPF1* expression peaked in the ileum and thymus at the 125 mg/ml dox dose, with slight decreases observed at the 500 mg/ml dose, whereas *dnUPF1* expression continued to increase in the spleen and white blood cells at the 500 mg/ml dox dose. Similar levels of gene expression were also observed when *Rpl13a* was used as the qPCR normalization control (Fig. S1A). These differences in *dnUPF1* tissue expression in response to dox could potentially be caused by a limitation of *rtTA* in certain tissues to complex with excess dox and induce transgene expression, or a limitation in the amount of dox penetrating certain tissues.

Because basal *dnUPF1* expression (−dox) was very low due to the tight regulation of the TET-ON expression system, the increase in *dnUPF1* expression upon dox treatment, as assayed by qPCR, might appear to be artificially elevated because we are comparing increases to a near-negligible background expression level (very high Ct values). To obtain a better estimate of *dnUPF1* expression, we also examined endogenous mouse *Upf1* and total *UPF1* expression (both transgene and mouse) (Fig. 2). We found small, but significant, less than 2-fold changes in mouse *Upf1* levels in the ileum and white blood cells from dox-treated mice. However, in the spleen (Fig. 2B), mouse *Upf1* levels became elevated up to 7-fold at the highest dox dose administered. These increases in endogenous *Upf1* expression are consistent with previous studies that observed certain NMD factors become elevated when NMD is inhibited due to a regulatory feedback mechanism (Huang et al., 2011). Quantitation of total *UPF1* indicated more modest increases than with *dnUPF1* quantitation (5- to 400-fold, depending on the tissue), most likely providing a more realistic indicator of overall *dnUPF1* expression levels. Consistent with *dnUPF1* expression, total *UPF1* in the ileum and thymus appeared to be maximally expressed at 125 mg/ml dox, while in the spleen and white blood cells, total *UPF1* expression continued to increase at 500 mg/ml dox. Overall, these data indicate that many mouse tissues express *dnUPF1* in response to dox, but the dox dose response appeared to differ among various tissues, which was mirrored by differences in *dnUPF1* expression.

**Fig. 3.**
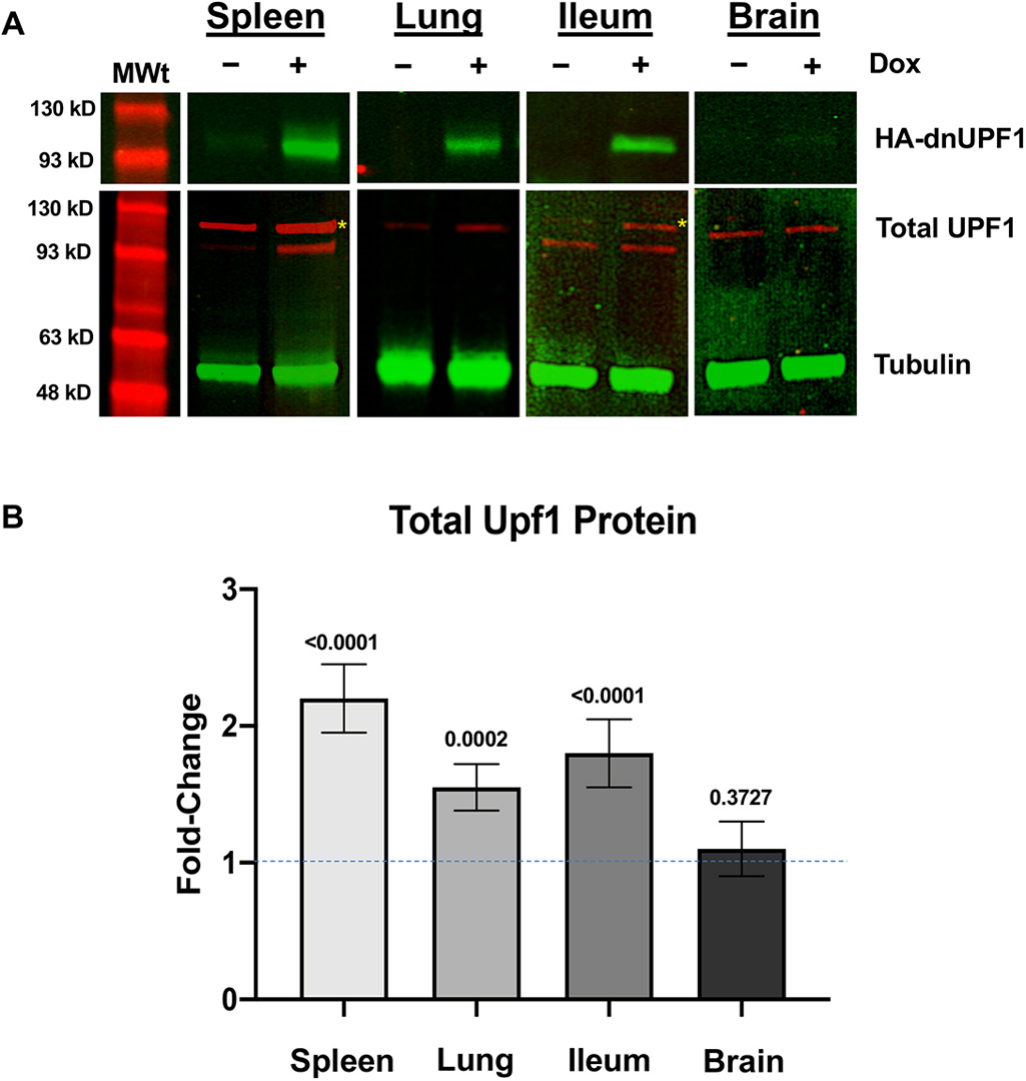
**Examination of dnUPF1 protein expression in transgenic mouse tissues.** (A) Representative western blot of dnUPF1 (green; antibody to HA epitope tag), total UPF1 (red; antibody to both human UPF1 and mouse Upf1), and tubulin (green; for normalization) in transgenic mice either vehicle alone or treated with 500 mg/ml doxycycline (dox) administration for 7 days. (B) Quantitation of total UPF1 protein by western blotting. Protein levels are expressed as the mean fold change (±s.d.) in protein levels from dox-treated mice relative to vehicle-treated mice (indicated by dashed line) and performed in triplicate from at least three mice per cohort. Exact *P*-values were calculated using an unpaired, two-tailed *t*-test and are shown in the figure. Note that multiple UPF1 bands are observed in some tissues, which is consistent with the presence of different isoforms due to alternative splicing. Yellow asterisks indicate the bands representing dnUPF1 in tissues with multiple isoforms.

We next verified dnUPF1 protein expression by immunoblotting mouse tissue lysates with antibodies to the HA epitope at the N terminus of dnUPF1. We found dnUPF1 protein was undetectable by western blotting in transgenic mouse tissues without dox administration (Fig. 3A), consistent with the low background expression of *dnUPF1*. However, administration of 500 mg/ml dox for 1 week induced robust expression of the dnUPF1 protein in all tissues examined except brain, where dnUPF1 expression was much less robust. Overall, dnUPF1 protein expression corresponded well with the RT-PCR data (Fig. 1B).

We next quantitated total UPF1 protein expression (normalized to tubulin) using an antibody that recognizes both the endogenous mouse Upf1 and the human UPF1 proteins. When blotting for total UPF1, multiple bands were observed in the spleen and ileum (Fig. 3A). The *dnUPF1* transgene is a cDNA and, therefore, is not subject to splicing. However, endogenous *UPF1* has been reported to be expressed as multiple isoforms in various tissues (Gowravaram et al., 2018). Thus, although some of these bands could potentially represent a nonspecific protein product, they most likely represent unique, tissue-specific UPF1 isoforms that may or may not also have differential post-translational modifications (Popp and Maquat, 2015). We observed only modest 1.1- to 2.5-fold increases in total UPF1 protein abundance (Fig. 3B). This result does not correlate with the large increases in *dnUPF1* mRNA expression observed in the spleen and ileum upon dox administration (Fig. 2). This observation at first seems consistent with a previous study that found that the abundance of many NMD factors is regulated by a negative feedback mechanism activated by NMD perturbation (Huang et al., 2011). mRNAs encoding many NMD factors carry a long 3′ UTR, making a subset of NMD factor transcripts subject to NMD. Perturbing NMD will therefore modulate the stability of certain NMD factor mRNAs to regulate their expression. However, the large differences between *dnUPF1* mRNA expression and protein expression cannot solely be explained by the NMD negative feedback mechanism. Furthermore, the *dnUPF1* transcript carries the short *SV40* 3′ UTR, not the long, endogenous *UPF1* 3′ UTR that renders these mRNAs subject to NMD. This suggests that other mechanisms might be controlling dnUPF1 protein abundance. Possibilities include: (1) reduced dnUPF1 protein stability when it is unable to complex with other NMD factors either due to its excess expression or due to a reduced binding affinity, or (2) reduced translation of the *dnUPF1* mRNA due to its expression from a cDNA that does not undergo splicing, which is known to enhance translation efficiency (Nott et al., 2004).

### Examining NMD efficiency in *dnUPF1* mice

**Fig. 4.**
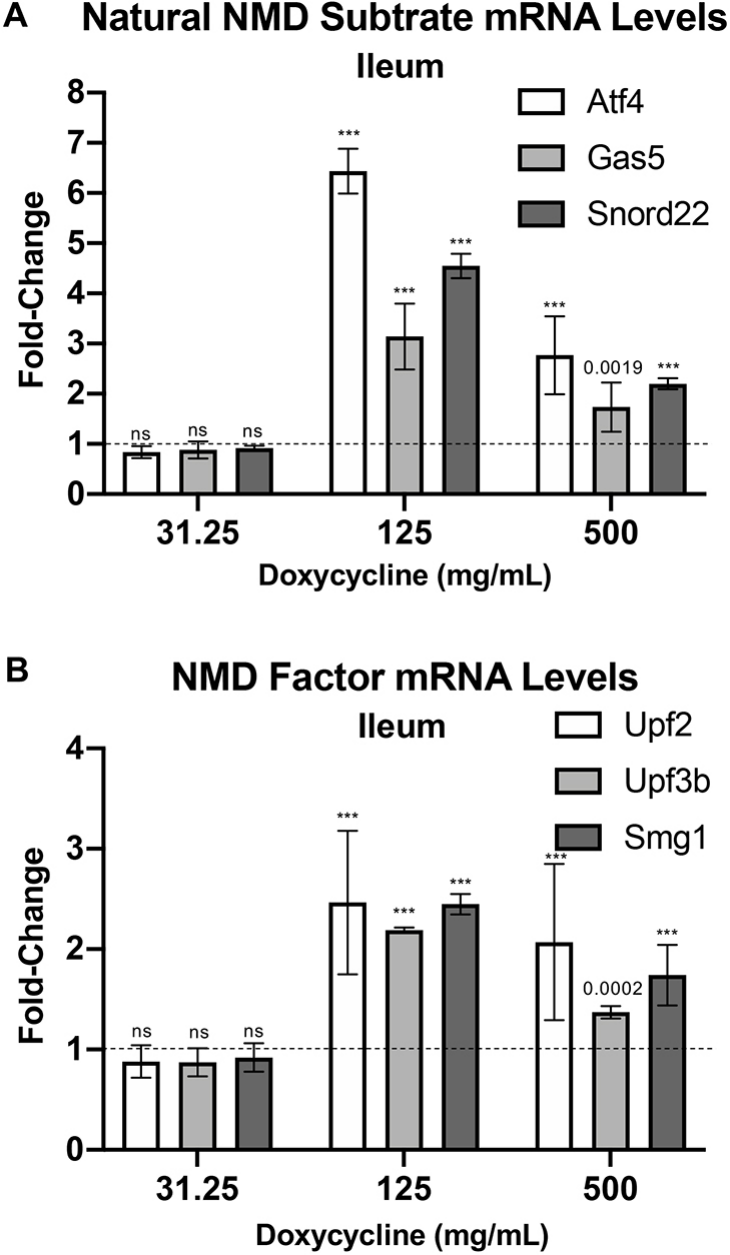
**dnUpf1 expression increases the steady state mRNA abundance of NMD substrates in the ileum of transgenic mice.** qPCR was performed to quantitate (A) endogenous NMD substrates *Atf4*, *Gas5*, and *Snord22* and (B) NMD factors *Upf2*, *Upf3b*, and *Smg1* in mouse ileum. The change in gene expression is expressed as the mean (±s.d.) fold change in steady state mRNA in transgenic mice treated with dox for 28 days relative to vehicle-alone controls (indicated by dashed lines). *n*=6 from at least three mice per cohort. *Gapdh* was used as a normalization control. Exact *P*-values were determined using an unpaired, two-tailed *t*-test comparing dox-treated mice to vehicle-only controls. ns, *P*>0.05; ***P<0.0001.

We next determined whether NMD could be attenuated upon *dnUPF1* expression. The NMD pathway impacts the expression of ∼10% of the mammalian transcriptome (Mendell et al., 2004). Physiological transcripts regulated by NMD include: transcripts containing uORFs in the 5′ UTR; transcripts with long or intron-containing 3′ UTRs; transcripts containing selenocysteine codons; and products of alternative splicing that harbor PTCs (Mendell et al., 2004). To evaluate the extent to which NMD is inhibited upon *dnUPF1* expression in the mouse ileum, we used qPCR to quantify the steady-state abundance of the natural NMD substrates, *Atf4*, *Gas5*, and *Snord22* in *dnUPF1* mice treated with different doses of dox for 28 days compared to vehicle-treated controls. *Atf4* encodes an ER stress and hypoxia-inducible transcription factor. The *Atf4* mRNA harbors uORFs that render it subject to NMD during non-stress conditions (Vattem and Wek, 2004). *Gas5* and *Snord22* are both translated small nucleolar host mRNAs that contain introns in their 3′ UTRs (Weischenfeldt et al., 2008). Following administration of dox, we found an increase in expression of all three NMD substrates when normalized to *Gapdh* (Fig. 4A) or to *Rpl13a* (Fig. S1B). At the level of highest NMD inhibition in the ileum, we observed a 3- to 6.5-fold increase in the abundance of these NMD substrates (Fig. 4A), which is comparable to the fold increase in NMD substrates previously reported with strong NMD inhibition in *Upf3b*-null mice (Huang et al., 2011; Shum et al., 2016). Taken together, these data suggest that we were able to inhibit NMD upon dox administration by inducing *dnUPF1* expression, and at the most effective dox concentration, a strong degree of NMD inhibition could likely be achieved in a at least a subset of tissues.

We also examined whether *dnUPF1* expression affected the abundance of other NMD factors, which have been shown to be NMD substrates that are negatively regulated by NMD and, thus, become elevated when NMD is inhibited (Huang et al., 2011). We observed a dox-mediated increase in mouse *Upf2*, *Upf3b*, and *Smg1* (Fig. 4B). At the maximum response, NMD factors were elevated 2- to 2.5-fold when normalized to either *Gapdh* (Fig. 4B) or to *Rpl13a* (Fig. S1C). Taken together, these data indicate that *dnUPF1* expression in these mice could be varied to inhibit NMD by different extents. Accordingly, this novel mouse serves as an appropriate model to better examine the effects of inhibiting NMD by different degrees *in vivo*.

### Characterizing *dnUPF1* mice

We next performed a thorough phenotypic characterization of *dnUPF1* mice administered different doses of dox beginning at 3 weeks (upon weaning), with treatment continuing for a period of 28 days. In this way, we could examine the effects of inhibiting NMD by different degrees on mammalian morphology and physiology.

We found that all dox-treated and vehicle-treated mice had a normal gross appearance. Although, from birth, *dnUPF1* mice weighed slightly less than wild-type (WT) mice (potentially from low-level *dnUPF1* expression during development), both *dnUPF1* and WT mice treated with dox gained weight at a similar rate over time (Fig. S2). Consistent with their slightly lower body weight, *dnUPF1* mice also had a slightly lower wet organ weight than WT controls (Fig. S3).

**Fig. 5.**
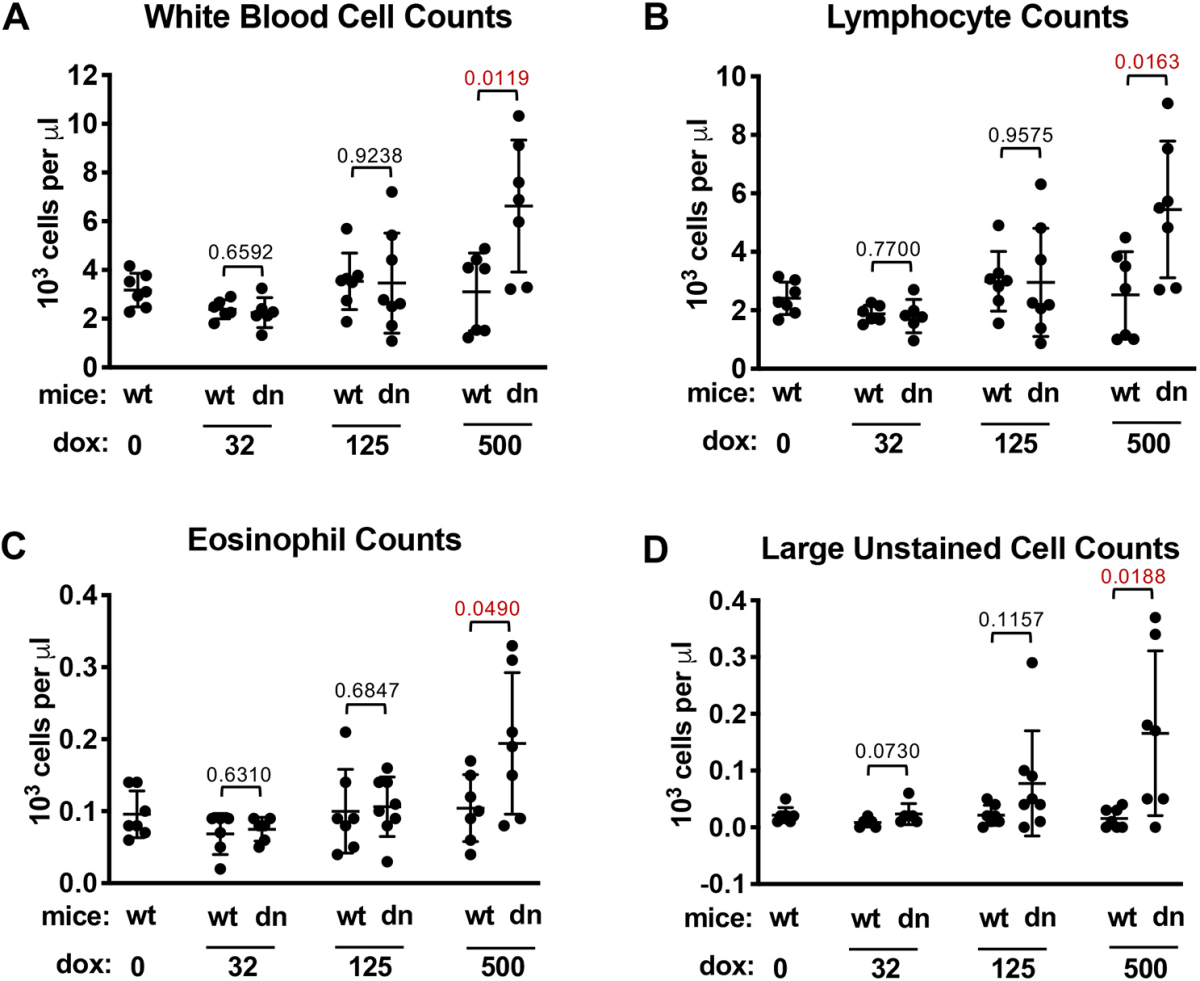
**Immunological assessment of *dnUPF1* mice.** A comprehensive blood count (CBC) was performed on blood samples from wild-type (wt) and *dnUPF1* (dn) mice treated with vehicle alone or dox at the indicated concentrations (mg/ml) for 28 days prior to analysis. Parameters in which significant differences were observed in dox-treated mice compared to controls include counts of (A) white blood cells, (B) lymphocytes, (C) eosinophils and (D) large unstained cells. The cell counts are presented as the mean (±s.d.) from 5-8 mice per cohort. Exact *P*-values were calculated using an unpaired, two-tailed *t*-test and are shown in the figure. All other CBC data are shown in Fig. S5.

A comprehensive histological assessment of male and female *dnUPF1* mouse tissues indicated normal morphology in all examined tissues and organs (Table S1). This is in contrast to a previous transgenic mouse model that constitutively expressed a *dnUPF1* transgene during prenatal development, resulting in failure to form a thymus (Frischmeyer-Guerrerio et al., 2011). In addition, examination of serum clinical chemistry did not reveal any endpoints for metabolism or for heart, liver or kidney functions outside of the normal range in either dox-treated or control mice (male or female) (Fig. S4). However, comprehensive blood counts (CBCs) revealed a significant increase in total white blood cells (Fig. 5A), lymphocytes (Fig. 5B), eosinophils (Fig. 5C) and large unstained cells (Fig. 5D) in *dnUPF1* mice administered the highest, 500 mg/ml dox dose, compared to vehicle controls. In contrast, no significant differences in these CBC endpoints were found in *dnUPF1* mice administered 125 mg/ml dox. In addition, no differences were found in additional CBC endpoints between *dnUPF1* and control mice (Fig. S5).

**Fig. 6.**
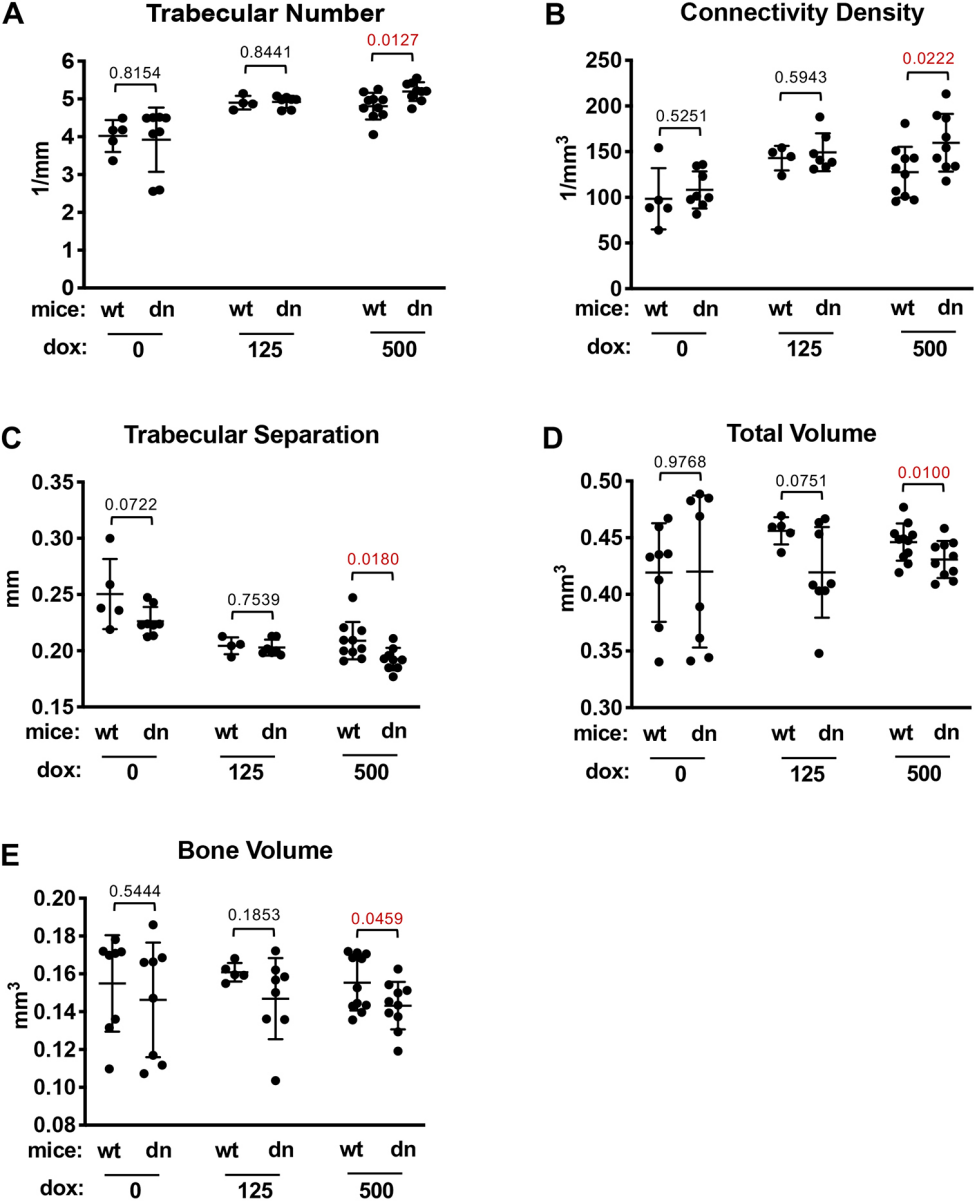
**Bone morphology assessment in female *dnUPF1* transgenic mice.** Micro-computed tomography was performed on the trabecular and cortical regions of femurs collected from wild-type (wt) and *dnUPF1* (dn) female mice treated with vehicle or dox at the indicated concentrations (mg/ml) for 28 days. Trabecular parameters that were found to be significantly different between some vehicle and 500 mg/ml dox cohorts include (A) trabecular number, (B) connectivity density and (C) trabecular separation. Cortical bone differences were found for (D) total tissue volume and (E) total bone volume. The values are presented as the mean (±s.d.) of the parameter calculated from 5-10 mice per cohort for each experimental condition. Exact *P*-values were determined using an unpaired, two-tailed *t*-test and are shown in the figure. All other bone assessment data are shown in Figs S6-S9.

Because immunological homing and maintenance can be linked to bone formation and morphology (Chamberlain et al., 2007; Stefanovic et al., 2013), we also examined the three-dimensional structure of trabecular and cortical bone in femurs from *dnUPF1* mice treated with dox compared to vehicle controls using micro-computed tomography. We found no aberrations in either trabecular (Fig. S6) or cortical bone (Fig. S7) parameters in dox-treated male mice. However, in female mice treated with the 500 mg/ml dox dose, we observed modest changes in the trabecular bone compared to WT controls, including an increased trabecular number (Fig. 6A), increased connectivity density (Fig. 6B) and decreased trabecular separation (Fig. 6C). In the female cortical bone, we found slight decreases in total volume (Fig. 6D) and bone volume (Fig. 6E). Together these parameters suggest a slight thickening of the femur in female mice when NMD was inhibited with the high dox dose. However, no additional trabecular (Fig. S8) or cortical (Fig. S9) bone parameters were affected in female mice treated with the high dox dose. Significantly, no aberrant trabecular or cortical bone structures were observed in female *dnUPF1* mice compared to controls when NMD was inhibited using a lower dox dose.

### Examining whether modest NMD inhibition enhances PTC suppression

From our *dnUPF1* mouse characterization, we found that mice treated at the highest, 500 mg/ml dox dose developed mild somatic defects. However, mice treated with 125 mg/ml dox or less did not develop any apparent abnormalities. This suggests that the 125 mg/ml dose likely inhibited NMD to a more modest extent in most tissues compared to the 500 mg/ml dose. We next wanted to determine whether the level of NMD attenuation observed with 125 mg/ml dox administration was adequate to increase the pool of PTC-containing mRNA and enhance PTC suppression.

**Fig. 7.**
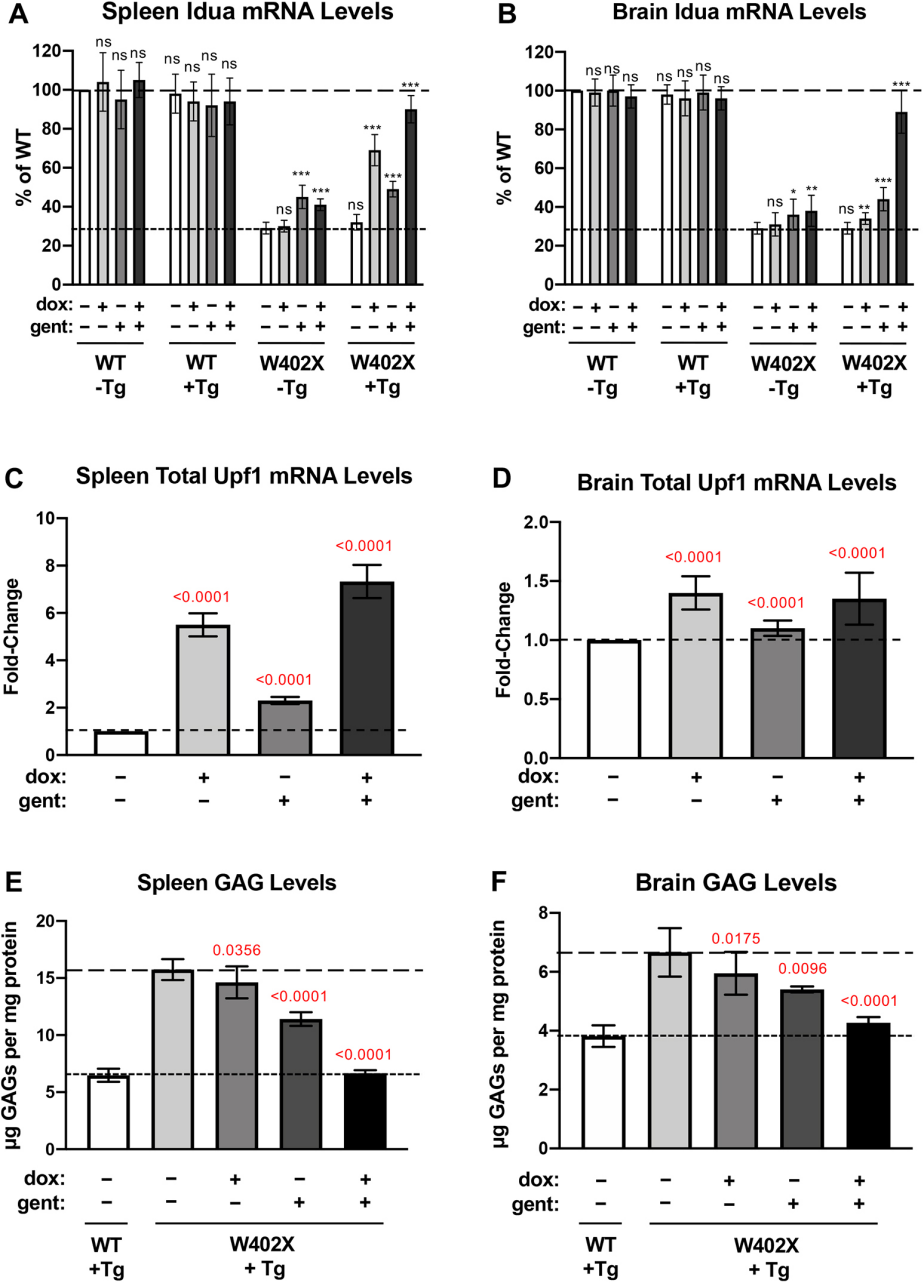
**Effect of *dnUPF1* expression on PTC suppression.** In a knock-in mouse model of MPS I-H that carries an *Idua-W402X* mutation and also expresses the *dnUPF1* transgene (Tg), *Idua* mRNA levels were examined in the (A) spleen and (B) brain of *dnUPF1/Idua-W402X* mice after treatment with vehicle or 125 mg/ml dox, and/or 30 mg/kg gentamicin (gent) for 14 days. Total *UPF1* mRNA levels were also examined in the (C) spleen and (D) brain of *dnUPF1/Idua-W402X* mice. Sulfated GAG levels were quantified in the (E) spleen and (F) brain of *dnUPF1/Idua-W402X* mice. In A and B, mean *Idua* mRNA levels (±s.d.) are expressed as the percentage of the level found in the untreated WT controls (no *dnUPF1* transgene, −Tg) (upper dashed line). The lower dashed line represents the average *Idua* mRNA abundance in untreated *Idua-W402X* mice (−Tg). Each condition was examined with *n*=6 from at least three mice per cohort. In C and D, total *UPF1* levels are expressed as the mean fold change (±s.d.) in treated mice relative to vehicle-alone controls (indicated by dashed lines), with three mice analyzed per group. In E and F, the mean sulfated glycosaminoglycan (GAG) levels (±s.d.) were calculated for each cohort of five mice. The lower dashed line indicates the WT GAG level, while the upper dashed line represents the GAG level in untreated mutant mice. Exact *P*-values comparing treated mice to vehicle controls were determined using an unpaired, two-tailed *t*-test and are shown in the figure for C-F. For A and B: ns, *P*>0.05; *P<0.01; **P<0.001; ***P<0.0001.

To perform this experiment, we utilized an *Idua-W402X* mouse model of mucopolysaccharidosis I-Hurler (MPS I-H). MPS I-H is a lysosomal storage disease that is caused by a severe deficiency of α-L-iduronidase, an enzyme that degrades a subset of glycosaminoglycans (GAGs). Without adequate α-L-iduronidase, undigested GAGs progressively accumulate in lysosomes, leading to onset of MPS I-H. MPS I-H is characterized by the formation of abnormalities in the joints, spleen, liver, heart and central nervous system. *Idua-W402X* knock-in mice carry a point mutation in the mouse *Idua* genomic locus that generates a PTC that is homologous to the human W402X mutation (Wang et al., 2010). Homozygous *Idua-W402X* mice express negligible levels of α-L-iduronidase, progressively accumulate GAGs and largely recapitulate most of the phenotypes observed in MPS I-H patients (Wang et al., 2010, 2012; Gunn et al., 2013; Keeling et al., 2013). Importantly, the W402X mutation triggers NMD of the *Idua* mRNA, significantly reducing its expression by 2- to 200-fold, depending on the tissue (Keeling et al., 2013). As shown in Fig. 7A,B, *Idua* mRNA abundance in both the spleen and brain of homozygous *Idua-W402X* mice is reduced to around 30-35% of WT levels. We crossed the *Idua-W402X* mice with *dnUPF1* transgenic mice to introduce the *dnUPF1* transgene under TET-ON control into MPS I-H mice. We used these *Idua-W402X/dnUPF1* mice to examine whether we could inhibit NMD of the *Idua-W402X* mRNA via *dnUPF1* expression and, if so, whether increases in *Idua-W402X* mRNA abundance achieved via moderate NMD attenuation enhance readthrough of the *Idua-W402X* mutation induced by the aminoglycoside, gentamicin.

*Idua-W402X/dnUPF1* mice were treated for 14 days with 125 mg/ml dox, a dose that inhibited NMD in *dnUPF1* transgenic mice without generating any overt morphological, biochemical or physiological abnormalities. In dox-treated *Idua-W402X/dnUPF1* mice, we found a 6-fold increase in total *UPF1* (mouse and *dnUPF1*) in the spleen compared to levels in controls (Fig. 7C), whereas a less than 2-fold increase was observed in the brain (Fig. 7D), which is consistent with our RT-PCR (Fig. 1B) and western blotting (Fig. 3) analyses of these tissues. This indicates that the level and pattern of *dnUPF1* expression in *Idua-W402X* mice is similar to the original transgenic *dnUPF1* strain.

We next examined *Idua* mRNA abundance in mouse spleen using qPCR (Fig. 7A). We found that gentamicin treatment alone led to an ∼1.5-fold increase in *Idua* mRNA in *Idua-W402X* mice with or without the *dnUPF1* transgene, which is in agreement with previous studies that showed some readthrough agents appear to stabilize PTC-containing mRNAs (Keeling et al., 2013). When *Idua-W402X/dnUPF1* mice were treated with dox alone to induce *dnUPF1* expression, a 2.5-fold increase in *Idua* mRNA was observed. Treating these mice with both dox and gentamicin resulted in a 3.2-fold increase in *Idua-W402X* mRNA, with *Idua* levels approaching those observed in WT mice.

In the brain of *Idua-W402X/dnUPF1* mice, increases in *Idua-W402X* mRNA abundance of less than 1.5-fold were observed in mice treated with either dox alone or gentamicin alone (Fig. 7B). However, when dox and gentamicin were co-administered to *Idua-W402X/dnUPF1* mice, a 3.6-fold increase in *Idua* mRNA abundance was observed, resulting in near normal *Idua* levels. This suggests that even though *dnUPF1* expression was induced at relatively low levels in the brain compared to the spleen at a 125 mg/ml dox dose, the low level of *dnUPF1* in the brain was still able to significantly inhibit NMD and increase *Idua* mRNA abundance.

We next examined whether the increases *in Idua-W402X* mRNA abundance via *dnUPF1* expression could enhance readthrough of the *Idua-W402X* nonsense mutation. Assaying α-L-iduronidase activity directly in tissue lysates using a fluorescent substrate is technically difficult due to high background fluorescence. We therefore monitored tissue GAG levels, which directly correlate with α-L-iduronidase activity, using a spectrophotometric dye-binding assay (Gunn et al., 2013; Keeling et al., 2001, 2013; Wang et al., 2012). We quantified GAG levels in the spleen and brain of *Idua-W402X/dnUPF1* mice treated with vehicle, gentamicin alone, dox alone or co-treated with both dox and gentamicin. We found that dox treatment alone slightly reduced GAG levels, suggesting that basal readthrough of an increased pool of *Idua-W402X* mRNA restores enough α-L-iduronidase function to modestly reduce GAG accumulation (Fig. 7E,F). When mice were treated with gentamicin, we observed a greater decrease in GAG accumulation due to an increase in readthrough of the *Idua-W402X* mutation. When we co-treated mice with dox and gentamicin, we saw GAG levels in both the spleen and brain return to WT levels. This suggests that a level of NMD attenuation that does not lead to deleterious phenotypes can be achieved to significantly enhance the amount of protein rescued by PTC suppression in somatic tissues.

## DISCUSSION

Although NMD represents a potential therapeutic target for rescuing protein function that is absent due to a PTC-forming mutation, concerns about the safety of inhibiting NMD have hindered its development as a therapeutic approach. One reason that the safety of attenuating NMD has not yet been resolved is a lack of suitable NMD-deficient animal models in which to examine the effects of reducing NMD efficiency *in vivo*. Genomic deletions of the NMD factors *Upf1* (Medghalchi et al., 2001), *Upf2* (Weischenfeldt et al., 2008) or *Smg1* (McIlwain et al., 2010) all result in embryonic lethality in mice and, thus, were unsuitable to examine NMD *in vivo*. More recently, viable NMD-deficient mice have been generated, but NMD was constitutively inhibited during development, resulting in the appearance of abnormalities. For example, *Upf3b*-null mice have been found to be viable, but these mice develop multiple neurological abnormalities (Huang et al., 2018). In addition, a viable transgenic mouse model that reduced NMD efficiency by expressing a dominant-negative *UPF1* transgene in a constitutive manner was previously described (Frischmeyer-Guerrerio et al., 2011). However, the constitutive expression of the mutant *UPF1* allele during embryonic development likely generated selective pressure for transgenic mice with weak NMD inhibition that permitted viability. A cursory morphological survey of these mice revealed faulty thymic development. However, no in-depth characterization was conducted to determine the extent of dominant-negative *UPF1* expression, the degree of NMD perturbation in other tissues or whether additional defects were present.

We hypothesized that a degree of NMD attenuation can be achieved after completion of prenatal development without the onset of harmful side effects. Because our strategy for generating inducible *dnUPF1* transgenic mice circumvented NMD inhibition during development, viable transgenic mice could be obtained without any selective pressure for weak NMD inhibition. This strategy permitted us to induce a wide range of *dnUPF1* expression in order to attenuate NMD efficiency by different degrees for various time periods. Unlike previous NMD-deficient mice, the tight, temporal regulation of *dnUPF1* expression in our transgenic mouse model allowed us to examine the effects of inhibiting NMD after completion of embryonic development, enabling us to more closely mimic the manner that global NMD attenuation might be implemented as a potential therapy *in vivo*.

Our *dnUPF1* mice broadly expressed the transgene in many organs and tissues, allowing us to access many phenotypic aspects of NMD inhibition. We were able to discern a dox-mediated increase in *dnUPF1* expression in multiple tissues that correlated with different degrees of NMD inhibition in many tissues that are comparable to the levels of NMD inhibition observed in other viable NMD-deficient mice (Huang et al., 2018). With the level of NMD inhibition achieved by dosing mice with 125 mg/ml dox, we found no morphological, physiological or biochemical aberrations in *dnUPF1* mice compared to controls. However, with the degree of NMD inhibition achieved by dosing *dnUPF1* mice with 500 mg/ml dox, we found modest elevations in subsets of blood cells. Previous studies have indicated that NMD plays a major role in the development of the immune system. Constitutive expression of a *dnUPF1* transgene was previously shown to abolish thymocyte development in mice (Frischmeyer-Guerrerio et al., 2011). In addition, NMD was found to be essential for the development of hematopoietic stem cells and progenitor cells in mice by participating in a quality control mechanism that eliminates non-productive products of programmed DNA rearrangements (Weischenfeldt et al., 2008). Because cells from multiple immunological lineages were affected in our *dnUPF1* mice during strong NMD inhibition, our data support the idea that NMD plays an important role in immunological homing and/or maintenance.

We also found that bone morphology became altered in female *dnUPF1* mice when NMD was inhibited by administering 500 mg/ml dox. Micro-computed topography data suggested a slight thickening of the femur in female mice, but no alterations of bone morphology were observed in male *dnUPF1* mice. This is the first evidence to suggest that NMD might play a role in bone growth and remodeling. Because this phenotype was only observed among female *dnUPF1* mice, it is likely that hormonal signaling pathways are also involved. Additional studies will be required to elucidate how NMD inhibition elicits this phenotype.

In addition, we found that a level of moderate global NMD attenuation that does not generate abnormal phenotypes in non-neurological, somatic tissues can significantly enhance the readthrough of PTCs and rescue physiologically significant levels of protein function. This suggests that a safe therapeutic window exists for inhibiting NMD in non-neurological, somatic tissues that might be effective for restoring deficient protein function in patients who carry a PTC.

NMD has been shown to play an important role in neurological development and function in humans (Tarpey et al., 2007; Nguyen et al., 2013; Jaffrey and Wilkinson, 2018) and in mice (Bokhari et al., 2018; Johnson et al., 2019). However, the mouse models that have been used to examine the neurological aberrations that arise during NMD inhibition, including a *Upf3b*-null mouse (Huang et al., 2018) and a conditional null mouse that lacks *Upf2* expression in the forebrain (Johnson et al., 2019), constitutively inhibit NMD during prenatal development. Thus, it has been unclear whether neurological dysfunction can be prevented if NMD is inhibited only after neonatal development. In this study, we found that, even after birth, the mouse brain appears to be more responsive to NMD perturbation than other organs, such as the spleen. This result suggests that NMD might function in a different manner in neurological tissues than in other somatic tissues.

Although the differential *dnUPF1* transgene expression prevents an exhaustive assessment of the effects of NMD inhibition in all tissues of *dnUPF1* mice, our initial characterization suggests that a moderate level of NMD inhibition that produces few harmful side effects is likely achievable in most non-neurological, somatic tissues. With the observation that neurological tissues appear to be hypersensitive to NMD attenuation, we cannot rule out the possibility that abnormalities in neurological tissues might develop when NMD is inhibited for long periods after prenatal development. Given that many pharmacological compounds cannot cross the blood/brain barrier, it is likely that drugs that modestly inhibit NMD without penetrating neurological tissues can be developed, abrogating any potential neurological side effects. Alternatively, a therapeutic approach that targets a specific branch of NMD or a specific NMD substrate rather than globally inhibiting NMD will likely further reduce the risk of deleterious side effects and may be also be used to target NMD substrates in neurological tissues.

## MATERIALS AND METHODS

### Generation of *dnUPF1* transgenic mice

The human *UPF1* cDNA containing an R844C mutation (Sun et al., 1998) was a gift from Lynn Maquat, University of Rochester, Rochester, NY, USA. It was subcloned into the EcoRI and NotI sites of pTRE-Tight (Clontech). Annealed primers (forward: 5′-GTACGCCTCCACGCTAGCGTAGTCTGGGACGTCGTATGGGTACATGGTGCCTCCG GGTAGGGCCCTCGGGCCGGTTCCGCGCTGCAG-3′ and reverse: 5′-CTAGCTGCAGCGCGGAACCG GCCCGAGGGCCCTACCCGGAGGCACCATGTACCCATACGACGTCCCAGACTACGCTAGCGTGGAGGC-3′) were used to fuse a hemagglutinin (HA) epitope tag to the N terminus of the construct (via EcoRI and BsiWI sites). The *HA-UPF1* construct was linearized using XhoI and the fragment containing the transgene was microinjected into C57BL/6 fertilized oocytes, which were implanted into pseudopregnant foster mothers to produce transgenic mice in the UAB Transgenic Mouse Core. Mice that carried the transgene, as identified by PCR, were bred with wild-type C57BL/6 mice, and progeny carrying the transgene were identified. These mice were crossed with *Gt(ROSA)26Sor*^tm1(rtTA,EGFP)Nagy^/J mice (Jackson Laboratory stock number 005572) that had been previously crossed with C57BL/6-Tg(Zp3-cre)93Knw/J mice (Jackson Laboratory stock number 003651) to excise the lox-flanked PGK-neo-pA fragment, allowing moderate, constitutive *rtTA* expression from the mouse *Rosa26* locus. Transgenic *dnUPF1* mice expressing the *rtTA* were bred to generate mice homozygous for both the *rtTA* (as indicated by PCR using the following primers: *rtTA/Rosa26* Pf, 5′-GAGTTCTCTGCTGCCTCCTG-3′; *rtTA* Pr, 5′-AAGACCGCGAAGAGTTTGTC-3′; *Ros26* Pr, 5′-CGAGGCGGATACAAGCAATA-3′) and *dnUPF1* as indicated by digital PCR (see below).

### Animal treatment

Doxycycline (Sigma; D9891) was dissolved in cherry-flavored sugar-free liquid Kool-Aid^®^, which was diluted 1:125 in sterile water. The doxycycline solution or Kool-Aid^®^ vehicle was administered to mice *ad libitum* in place of their regular drinking water. Doxycycline administration ranged from 7 to 28 days at concentrations that ranged from 31.25 mg/ml to 1000 mg/ml. For 7-day dox dosing, treatment was initiated in 8-week-old mice. For 28-day dox dosing, treatment was initiated in 3-week-old mice. At the end of treatment, blood was collected from the animals via cardiac puncture followed by perfusion with cold PBS. Tissues were harvested, flash frozen, and stored at −80°C until assayed. To induce readthrough in *Idua-W402X* mice (Wang et al., 2010) expressing the *dnUPF1* transgene, gentamicin (VetOne, Boise, ID, USA) was administered once daily via subcutaneous injections at a dose of 30 mg/kg for 14 days. Both male and female mice were used in all analyses. All animal work was conducted according to relevant national and international guidelines and all animal protocols used in this study were reviewed and approved by the UAB IACUC (protocol numbers IACUC-20132 and IACUC-20261).

### Determination of transgene copy number

Digital PCR was performed using the QuantStudio 3D Digital PCR System with the QuantStudio 3D Digital PCR 20K Chip Kit v2 (ThermoFisher; A26316). Briefly, the *dnUPF1* transgene was PCR amplified in a 36 µl reaction (2 chips: 1 experimental and 1 reference) containing QuantStudio 3D Digital PCR Master Mix SYBR Green Supermix (Bio-Rad) and TaqMan Probes specifically to the *dnUPF1* transgene or to a TaqMan copy number reference, mouse *Tfrc* (ThermoFisher; 4458366). The PCR reaction was carried out as follows: 96°C for 10 min, followed by 60°C for 2 min, and then 39 cycles of 98°C for 30 s and 60°C for 2 min. Data analysis was performed using the QuantStudio 3D Analysis Suite Cloud Software (ThermoFisher).

### RT-PCR

Total RNA was isolated from mouse tissues using an Ambion RiboPure kit followed by DNAase treatment (Ambion Turbo DNA-free kit). Polyadenylated RNA was reverse transcribed into cDNA in a 50 µl reaction containing 1 µg of total RNA, 0.5 mg/ml oligo dT, 1.2 mM dNTPs, 40 U RNasin (Promega), 10 µl of 5× AMV RT buffer and 40 U AMV reverse transcriptase (Promega).

RT reactions were incubated at 42°C for 1.5 h, and then heat inactivated at 65°C for 15 min. The cDNA was ethanol precipitated and resuspended in 50 µl of 10 mM TRIS, pH 8.0, containing 0.1 mM EDTA. Equal amounts of cDNA (10% of the total cDNA) were used to perform PCR using the following thermocycler conditions: 95°C for 5 min followed by 35 cycles of 30 s at 95°C, 45 s at 62°C, 1 min at 72°C, followed by 10 min at 72°C and then stored at 4°C. The following primers sets (forward, Pf; reverse, Pr) were used: *dnUPF1* Pf, 5′-CGCCTGGAGAATTCATGTACCC-3′ and *dnUPF1* Pr, 5′-AACTCGAACTCGGAGCCCTG-3′; *rtTA* Pf, 5′-GAGTTCTCTGCTGCCTCCTG-3′ and *rtTA* Pr, 5′-AAGACCGCGAAGAGTTTGTC-3′.

### Quantitative RT-PCR

qPCR was performed in a 25 µl reaction containing 12.5 µl iQ SYBR Green Supermix (Bio-Rad), 0.2 µM of each forward and reverse primer, and cDNA (2 µg for each transcript). The following primers sets (forward, Pf; reverse, Pr) were used: *Idua* Pf, 5′-TGACAATGCCTTCCTGAGCTACCA-3′ and *Idua* Pr, 5′-TGACTGTGAGTACTGGCTTTCGCA-3′; *Atf4* Pf, 5′-CACAACATGACCGAGATGAG-3′ and *Atf4* Pr, 5′-CGAAGTCAAACTCTTTCAGATCC-3′; *Gas5* Pf, 5′-TTTCCGGTCCTTCATTCTGA-3′ and *Gas5* Pr, 5′-TCTTCTATTTGAGCCTCCATCCA-3′; *Snord22* Pf, 5′-GCCAGGCCTGTTCAATTTTA-3′ and *Snord22* Pr, 5′-TGCCTGAGATTTGTCACCAG-3′; *Gapdh* Pf, 5′-TTCCAGTATGACTCCACTCACGG-3′ and *Gapdh* Pr, 5′-TGAAGACACCAGTAGACTCCACGAC-3′; *Rpl13a* Pf, 5′-ATGACAAGAAAAAGCGGATG-3′ and *Rpl13a* Pr, 5′-CTTTTCTGCCTGTTTCCGTA-3′; Mouse *Upf1* Pf, 5′- GTACTTCCAGACCCATGACCAGATCA-3′ and Mouse *Upf1* Pr, 5′-CCCCACGGCCAGTCTTGGT-3′; Total *UPF1* Pf, 5′-CTGGAGGACCTGGAGAAGCC-3′ and Total *UPF1* Pr, 5′-CGGGTGATGTTATCTTGAGTCTG-3′; *dnUPF1* Pf, 5′-CGCCTGGAGAATTCATGTACCC-3′ and *dnUPF1* Pr, 5′-AACTCGAACTCGGAGCCCTG-3′; mouse *Upf2* Pf, 5′-TGCTAAGACCAAAGATCAAACTC-3′ and mouse *Upf2* Pr, 5′-CTCCTCCTCAGAACCCTCTT-3′; mouse *Upf3b* Pf, 5′-CAGGGACCGATTTGATGG-3′ and mouse *Upf3b* Pr, 5′-CTCATTGTCTGTGGCATAACTCTC-3′; mouse *Smg1* Pf, 5′- GACCA GCCTA CAATC CATCC-3′ and mouse *Smg1* Pr, 5′-CAAAC TCTGC AACCA CCCA-3′. qPCR was performed with the CFX96 Real-Time PCR Detection System (Bio-Rad) using a program that included an initial 3-min denaturation step at 95°C followed by 40 repeated cycles of a 10 s denaturation step at 95°C and a 30 s annealing/extension step at 55°C. Melt curve analysis was initially performed with each primer set to verify that only one gene product was generated from the PCR reactions. A standard curve was performed using each primer set to ensure that, under the PCR conditions used, the efficiency ranged between 90 and 110%. The average quantification cycle (Cq) was determined for each mRNA, and mRNA quantification was performed using the Livak (ΔΔCq) method (Livak and Schmittgen, 2001) where *Gapdh* or *Rpl13a* served as normalization controls. Cq values among the different samples for the various transcripts ranged from 8 to 30. qPCR was performed using at least 8-12 replicates for each gene product from each sample.

### Western blotting

Fifty micrograms of total protein for each lysate was subjected to SDS-PAGE and transferred to Immobilon-P membrane (Millipore). Blots were incubated with HA (Covance; 901515) or UPF1 (Abcam; ab109363) antibodies according to manufacturer instructions. Tubulin was used as an internal control and detected using a mouse monoclonal antibody (Developmental Studies Hybridoma Bank at the University of Iowa; E7). LI-COR goat anti-mouse IgG (LI-COR Biosciences; 925-32210) and goat anti-rabbit IgG (H+L) (LI-COR Biosciences; 925-32211) are secondary antibodies that were used to visualize protein bands, which were detected and quantitated using the LI-COR Odyssey Clx Imaging System (LI-COR Biosciences).

### Histology

Histology was performed by the UAB Comparative Pathology Laboratory. Mice were sacrificed by exsanguination under deep anesthesia induced by intraperitoneal injection of 100 mg/kg pentobarbital. Liver, spleen, kidneys, heart, lungs and brain were collected and fixed in alcoholic formalin containing 75% ethanol and 10% formalin. Tissues were then rinsed and stored in 10% neutral buffered formalin or buffered ethanolic formalin containing 75% ethanol and 10% formalin. Tissues were trimmed thus: liver, center of the left lobe and right portion of the median lobe; spleen, hemi-sectioned longitudinally; kidneys, hemi-sectioned longitudinally through the pelvis; heart, hemi-sectioned through the long axis and perpendicular to the septum; lung, multiple cross sections across all lobes; and brain, entire organ cut into 2 mm blocks, including cuts at the optic chiasm and the midpoint of the cerebellum. Tissues were processed routinely for paraffin sectioning, sectioned at 5 µm thickness and stained with Hematoxylin and Eosin (HE). Duplicate sections were stained with periodic acid-Schiff and Hematoxylin (PASH). Selected tissues were digitally photographed using a Nikon E600 microscope and SPOT Insight^®^ digital camera (Diagnostic Instruments, Inc., Sterling Heights, MI, USA).

### Clinical chemistry

Whole blood was collected on the final day of treatment in untreated tubes and allowed to clot at room temperature for 30 min. Serum was then collected in clean untreated tubes following centrifugation at 2000 RPM (376 ***g***) for 15 min and stored at 4°C until assay. A complete comprehensive panel was performed by Charles River Clinical Pathology Services, Shrewbury, MA. USA.

### CBCs

Whole blood was collected on the final day of treatment in EDTA-coated tubes and stored at 4°C until assay. A complete blood count/differential was performed by Charles River Clinical Pathology Services, Shrewbury, MA, USA.

### Micro-computed tomography

Excised femurs from mice were scanned using the Scanco µCT40 desktop cone-beam micro-CT scanner (Scanco Medical AG, Brüttisellen, Switzerland; using µCT Tomography v5.44). The femur was placed inverted in a 12 mm diameter scanning holder and scanned at the following settings: 12 µm resolution, 70 kVp, 114 µA, 500 projections/180 degrees with an integration time of 200 ms. Scans were automatically reconstructed into 2D slices and all slices were analyzed using the µCT Evaluation Program (v.6.5-2, Scanco Medical). 3D images were obtained from the 3D evaluation software (µCT Ray v.3.8, Scanco Medical). For the cortical analysis, the bone was scanned at the midshaft of the bone for a scan of 25 slices. The region of interest (ROI) was drawn on every slice and fitted to the outside of the cortical bone, to include all the bone and marrow. The threshold for cortical bone was set at 1033 mgHA/cm^3^. The 3D reconstruction (µCT Ray v3.8) was performed using all the outlined slices. Data were obtained on bone volume (BV), total volume (TV), BV/TV, bone density and cortical thickness. For the trabecular bone, the scan was started at the growth plate and consisted of 210 slices. The ROI was outlined starting 20 slices below the growth plate. 100 slices were outlined from this point, on the inside of the cortical bone, enclosing only the trabecular bone and marrow. Trabecular bone was thresholded at 547 mgHA/cm^3^ and the 3D analysis performed on the 100 slices. Data were obtained on bone volume, density, total volume, trabecular number, thickness and separation.

### GAG assay

This assay was performed as previously described (Gunn et al., 2013; Keeling et al., 2013; Wang et al., 2012). Tissues were homogenized using a Tissue Tearor homogenizer in chloroform:methanol (2:1 v/v). Defatted tissue was dried in a SpeedVac and then suspended in 100 mM dibasic sodium phosphate, pH 6.5, containing 0.6 mg/ml cysteine and 2 mg/ml papain (Sigma; P4762). The mixture was digested at 60°C for 18-24 h with constant agitation. The samples were then microfuged at 10,000 ***g*** for 15 min, and the supernatant was used to quantitate the tissue GAGs using the Blyscan Sulfated GAG Assay (Biocolor, UK). The total amount of sulfated GAGs precipitated from each sample was determined from a standard curve using chondroitin 4-sulfate (Sigma; C9819). The GAG levels are expressed as micrograms of GAGs per milligram of defatted, dried tissue.

### Statistics

All statistics were calculated with unpaired, two-tailed *t*-tests using GraphPad Prism software.

## Supplementary Material

Supplementary information
