# Supplementary material for: A regulated NMD mouse model supports NMD inhibition as a viable therapeutic option to treat genetic diseases
Source: Dis Model Mech. 2020 Aug 27;13(8):dmm044891. doi: 10.1242/dmm.044891 (PMC7473645; doi:10.1242/dmm.044891)
Supplement: Supplementary information [file dmm-13-044891-s1.pdf]

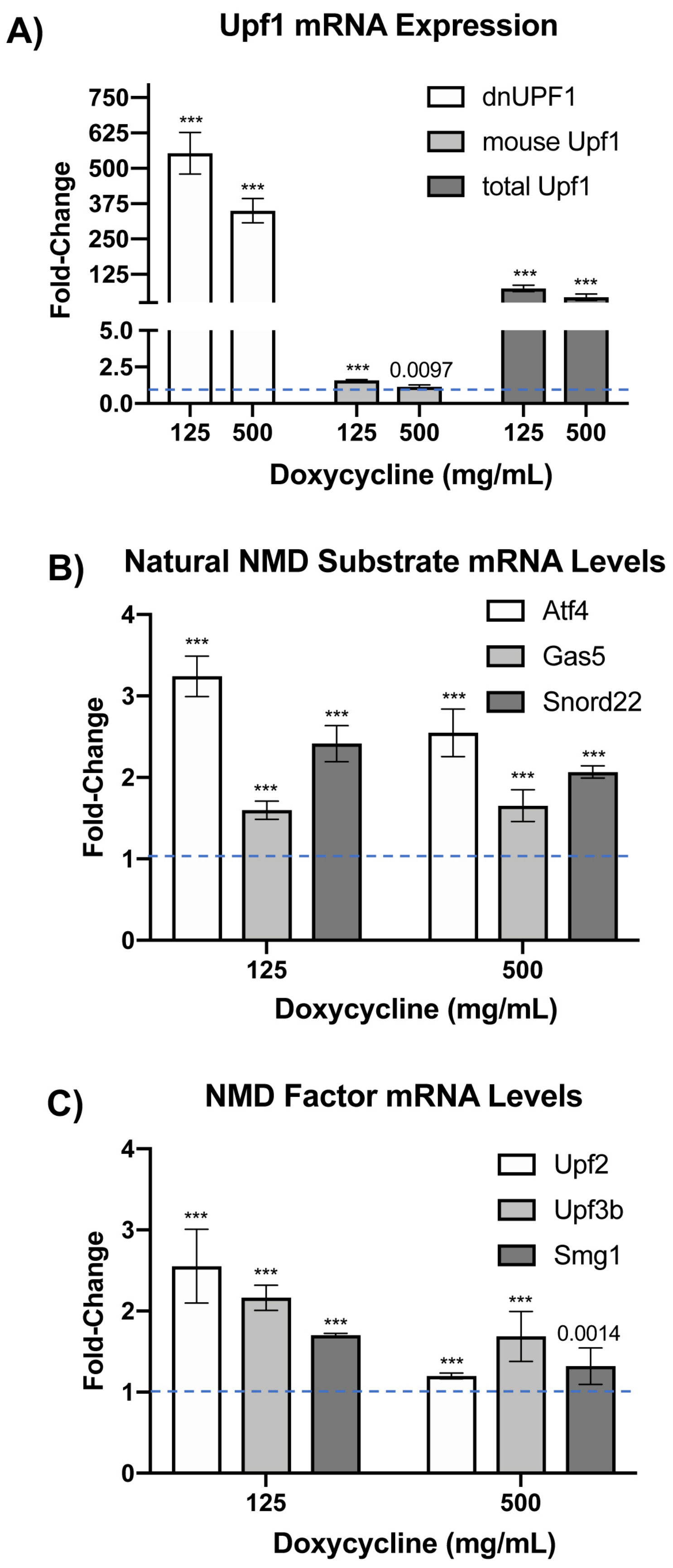

Fig. S1: *Quantification of mRNA levels in the ileum of dnUPF1 mice.* qPCR was used to quantify: **A)** Ufp1, **B)** NMD substrate, and **C)** NMD factor mRNA abundance. The data is the mean fold-change (+/- SD) in steady state mRNA in mice treated with doxycycline for 28 days relative to vehicle-alone controls (=1; dashed line) after normalization to *Rpl13a*. Three mice are included in each cohort. Exact p values were determined by comparing dox-treated mice to the vehicle control using a unpaired t-test; *** indicates p<0.0001.

Disease Models & Mechanisms • Supplementary information

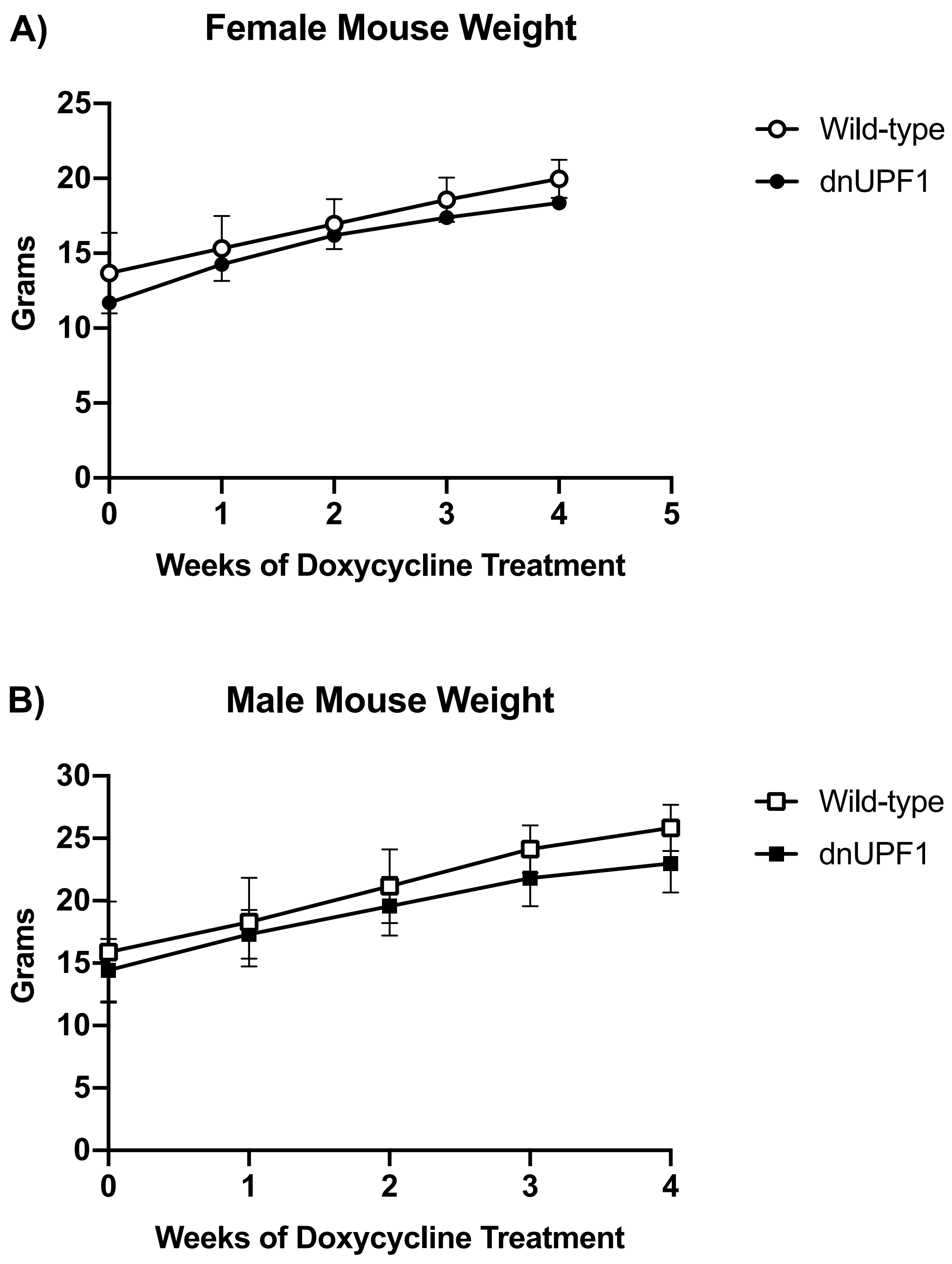

Fig. S2: *Mouse weight during treatment with 500 mg/mL doxycycline for 28 days.* Each point represents the mean (+/- SD) weight of 43-57 mice per cohort.

Disease Models & Mechanisms • Supplementary information

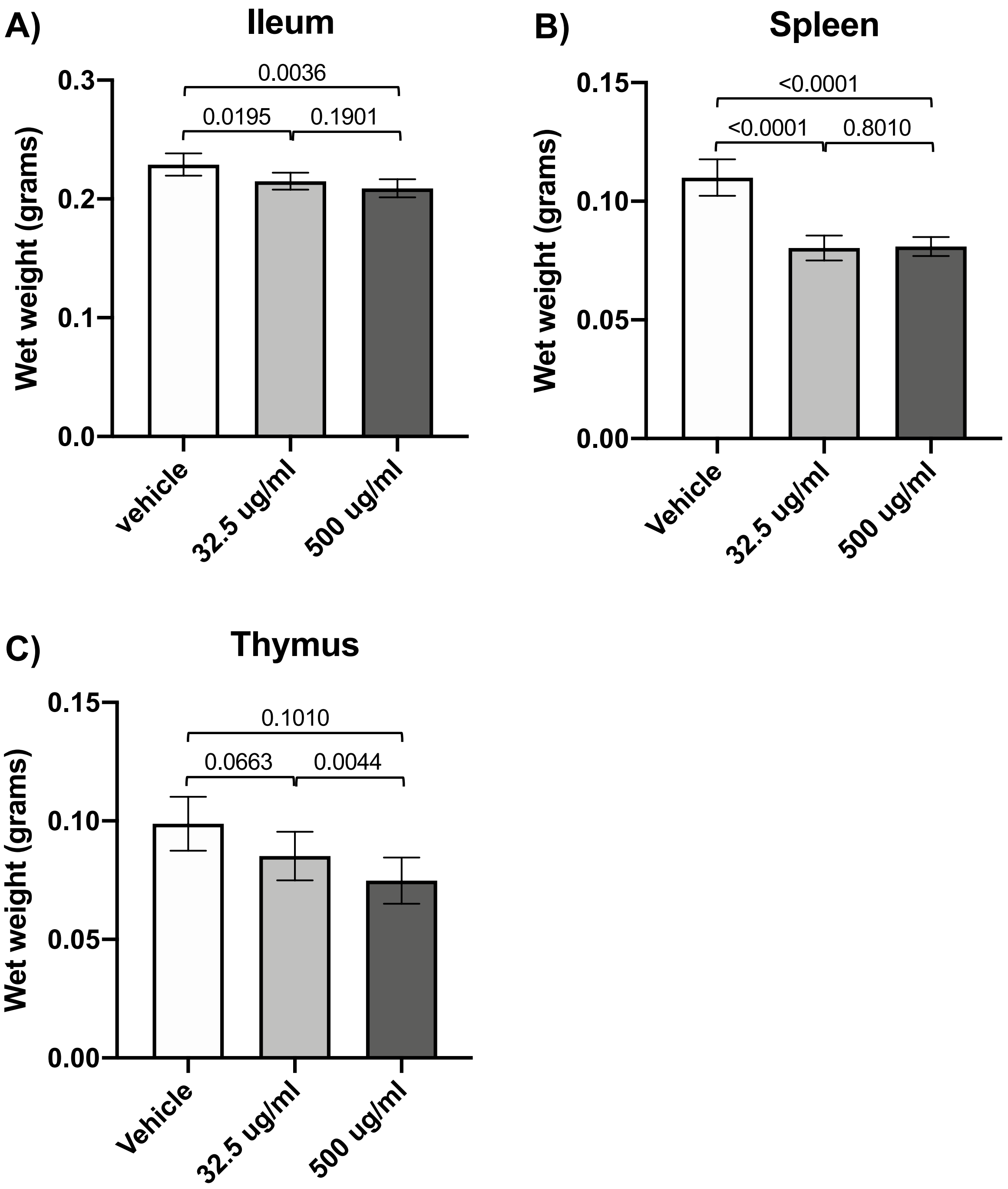

Fig. S3: *Wet tissue weight.* Each bar represents the mean (+/- SD) dnUPF1 tissue weights after 28 days of vehicle or doxycycline administration. p values were calculated using an unpaired t-test, where six mice were in each cohort.

Disease Models & Mechanisms • Supplementary information

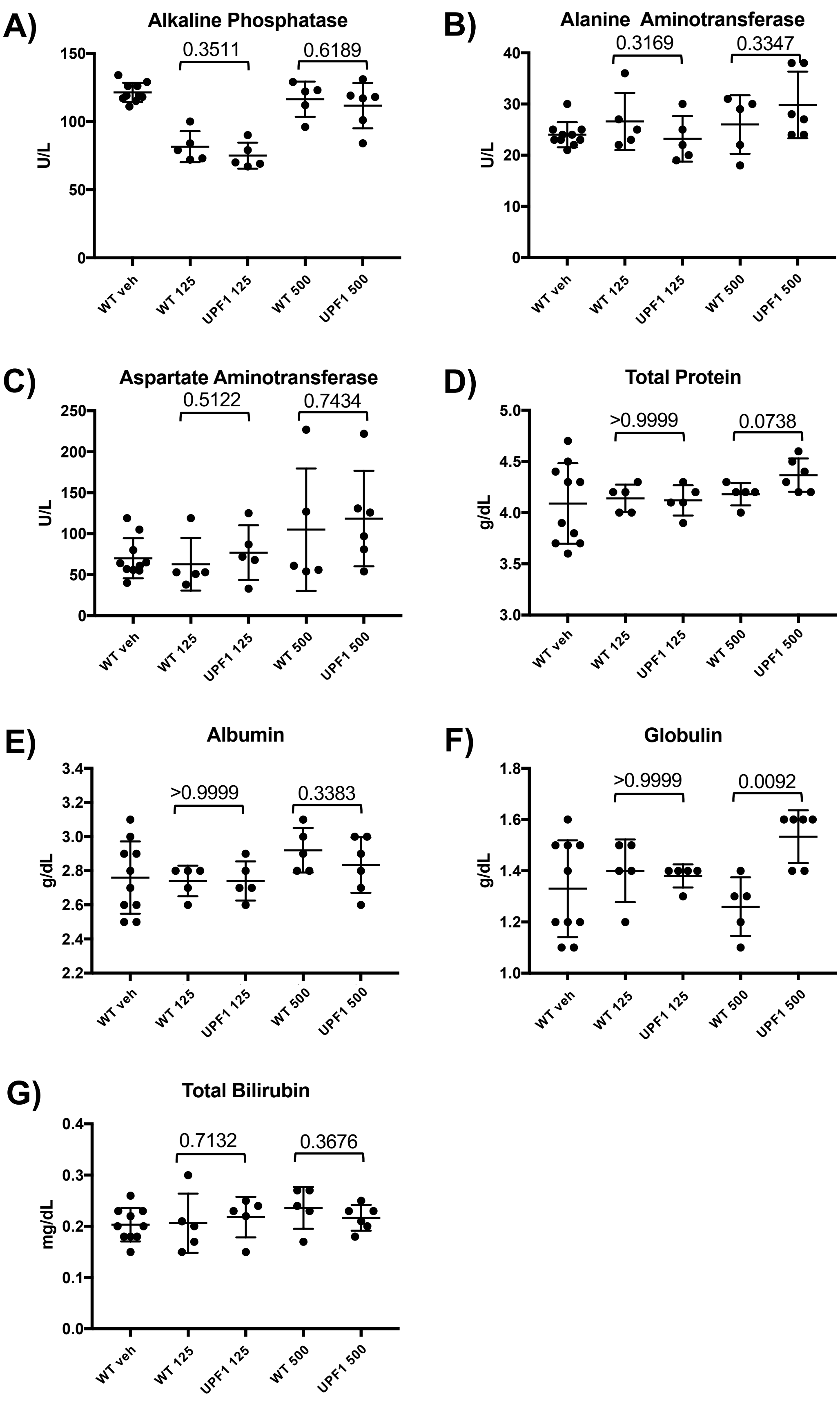

Disease Models & Mechanisms • Supplementary information

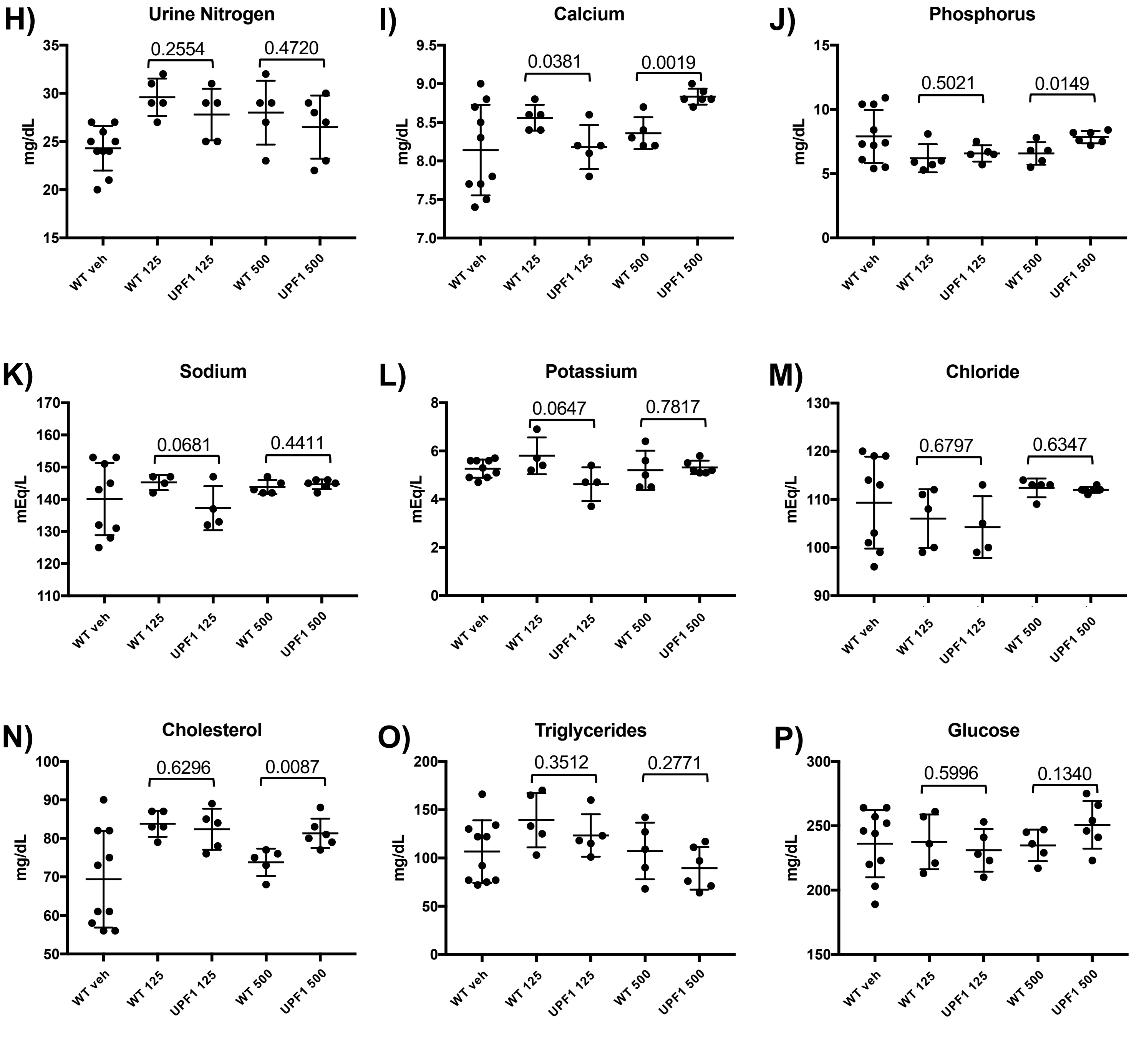

Fig. S4: *Clinical chemistry assessment of dnUPF1 mice.* WT or dnUPF1 mice were treated with vehicle or doxycycline (mg/mL) for 28 days. Blood samples were analyzed for various heart, liver, and kidney functional markers. Exact p values were calculated using an unpaired t-test, with each cohort containing 5-10 mice.

Disease Models & Mechanisms • Supplementary information

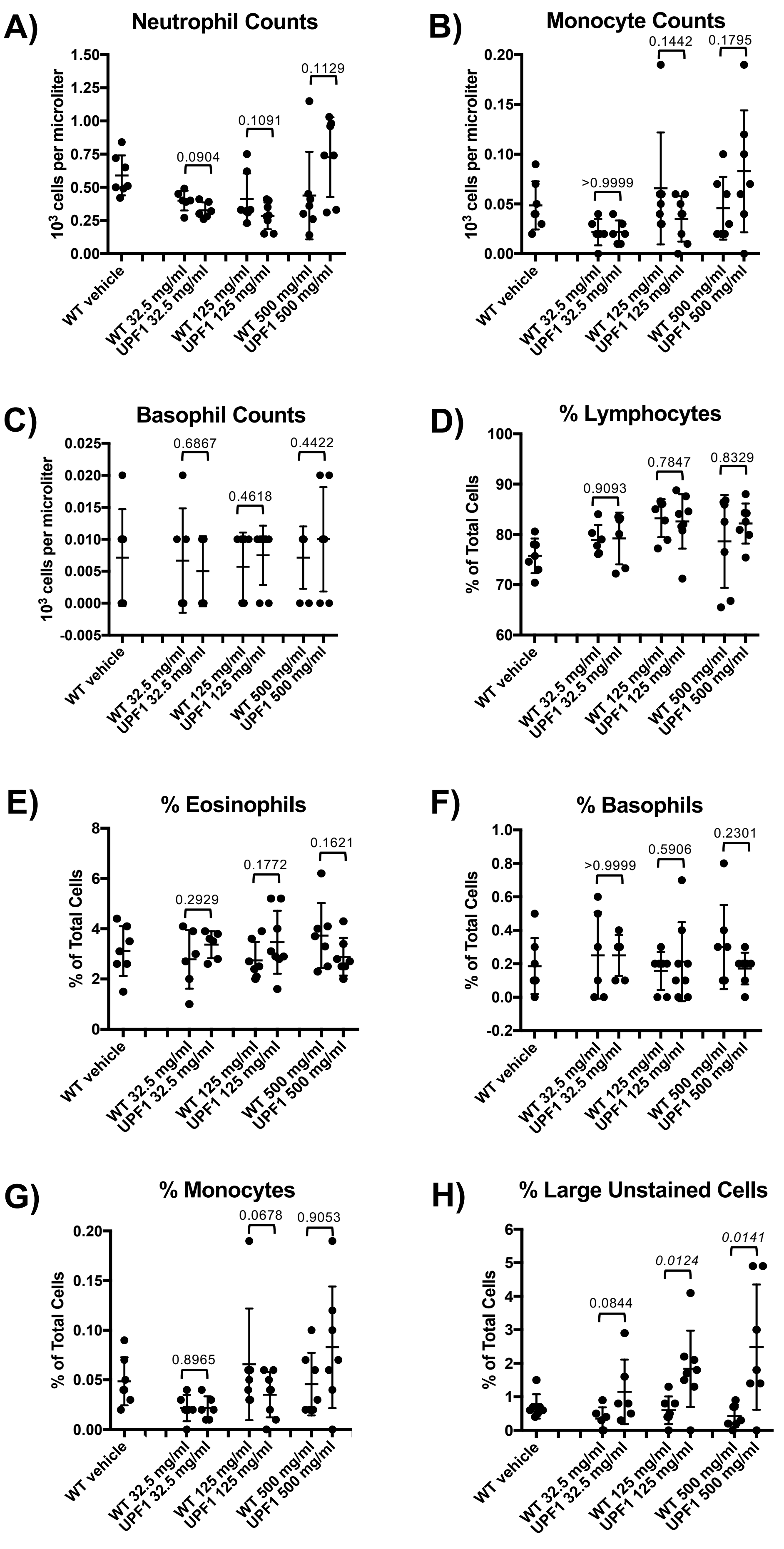

Disease Models & Mechanisms • Supplementary information

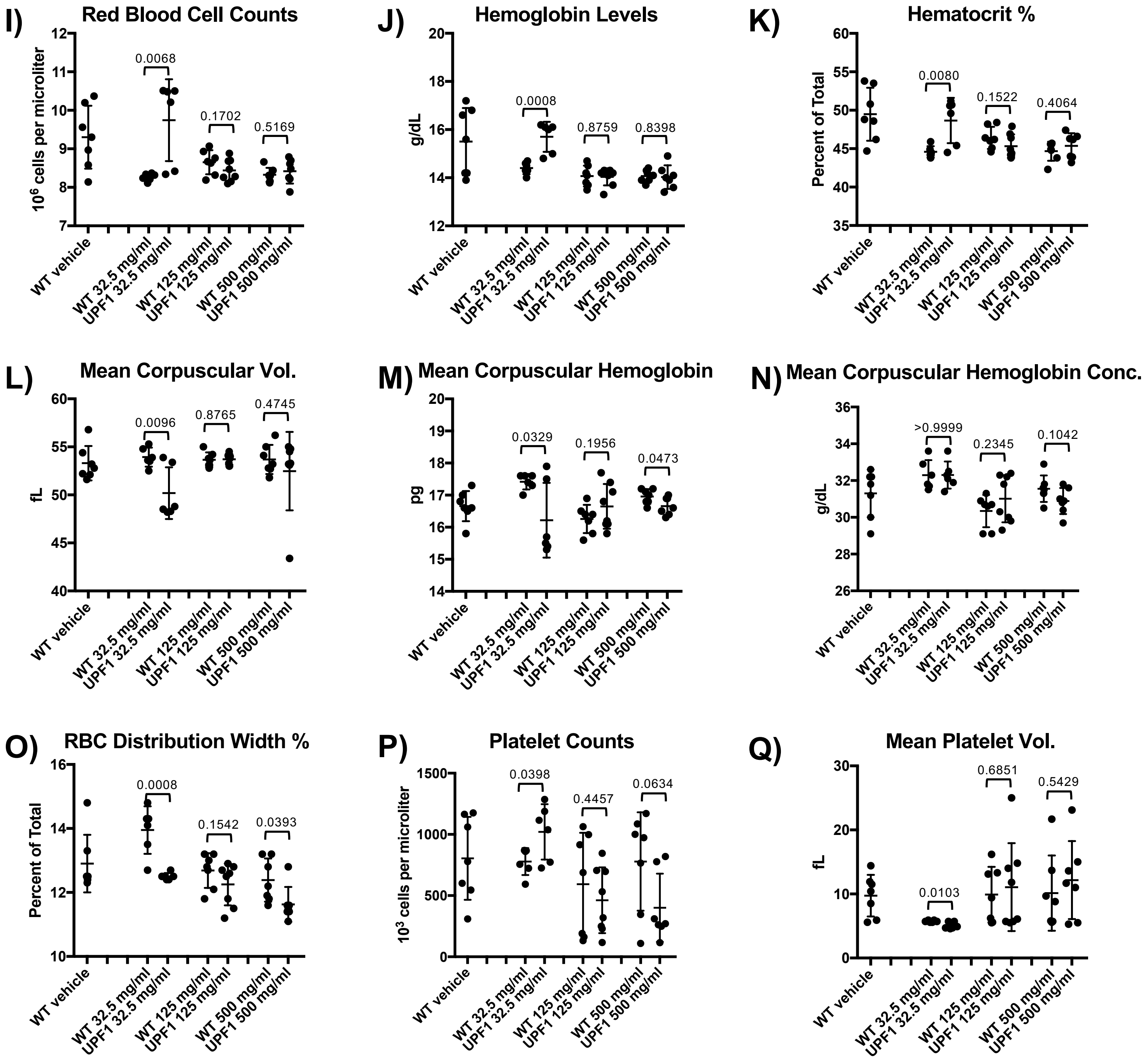

Fig. S5: *Comprehensive blood counts in WT or dnUPF1 transgenic mice treated with vehicle or doxycycline for 28 days.* The cell counts are presented as the mean (+/- SD) cell counts from 5-8 mice per cohort. Exact p values were calculated using an unpaired t-test.

Disease Models & Mechanisms • Supplementary information

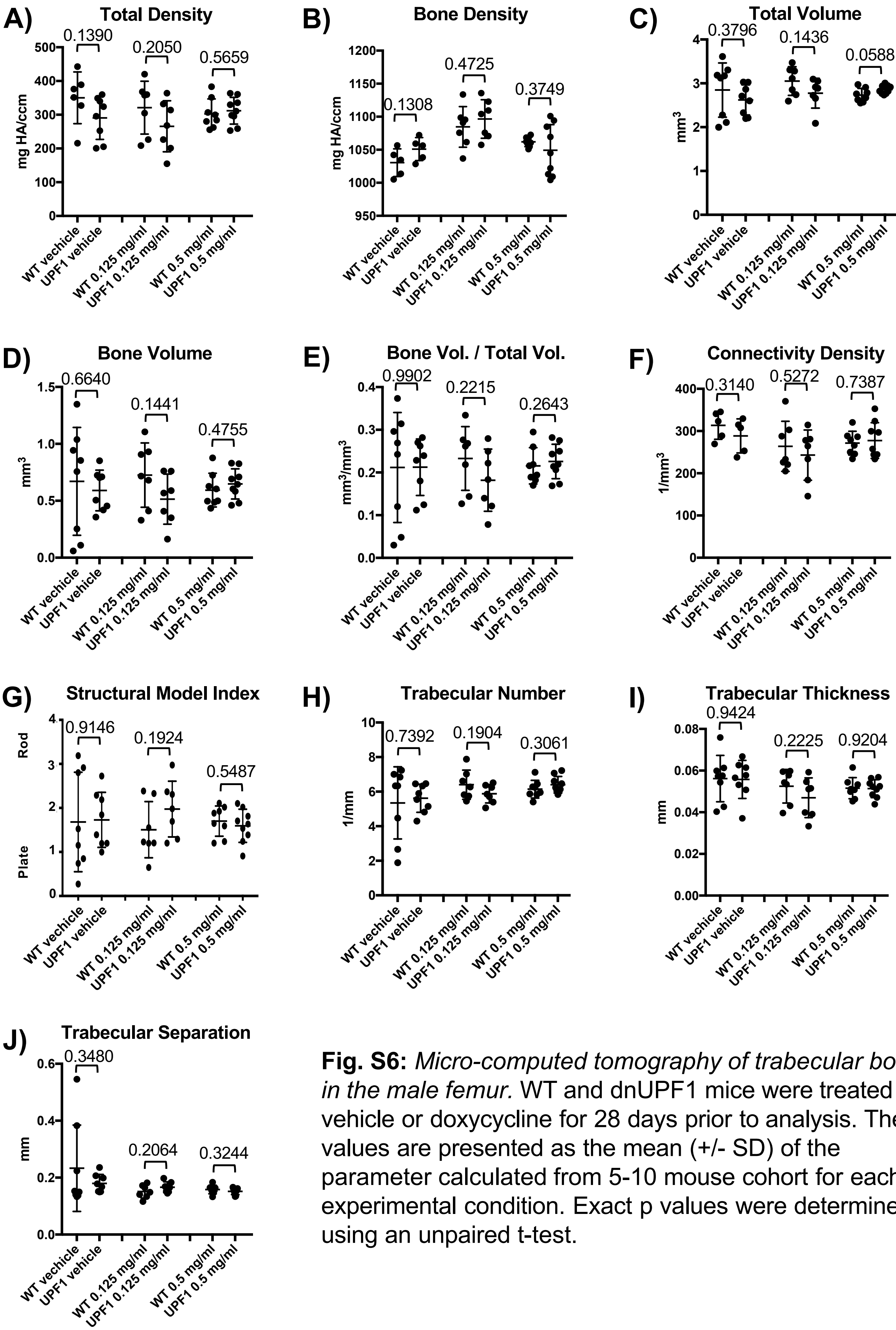

Fig. S6: *Micro-computed tomography of trabecular bone in the male femur.* WT and dnUPF1 mice were treated with vehicle or doxycycline for 28 days prior to analysis. The values are presented as the mean (+/- SD) of the parameter calculated from 5-10 mouse cohort for each experimental condition. Exact p values were determined using an unpaired t-test.

Disease Models & Mechanisms • Supplementary information

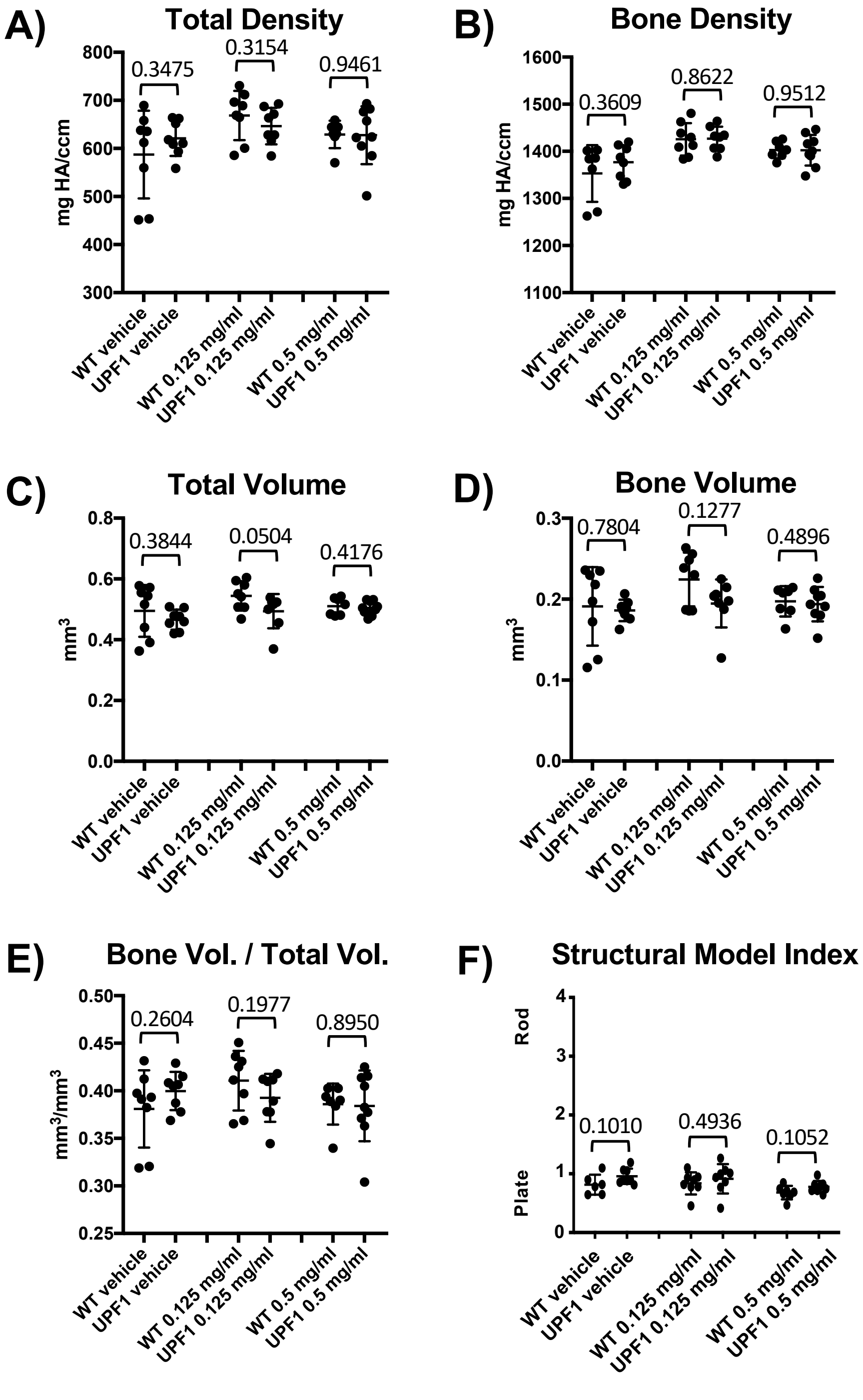

Fig. S7: *Micro-computed tomography of cortical bone in the male femur.* WT and dnUPF1 mice were treated with vehicle or doxycycline for 28 days prior to analysis. The values are presented as the mean (+/- SD) of the parameter calculated from 5-10 mouse cohort for each experimental condition. Exact p values were determined using an unpaired t-test.

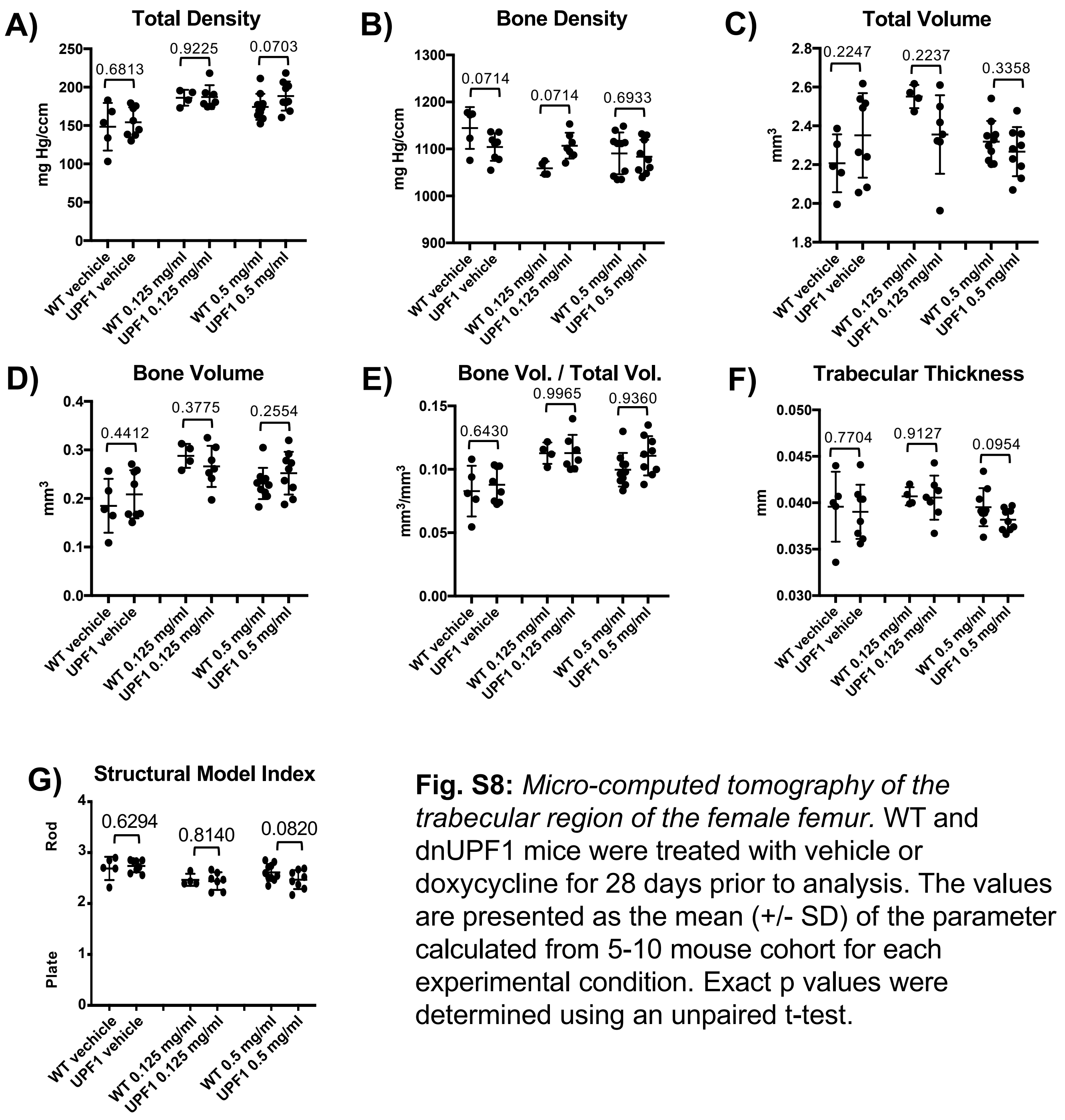

Fig. S8: *Micro-computed tomography of the trabecular region of the female femur.* WT and dnUPF1 mice were treated with vehicle or doxycycline for 28 days prior to analysis. The values are presented as the mean (+/- SD) of the parameter calculated from 5-10 mouse cohort for each experimental condition. Exact p values were determined using an unpaired t-test.

Disease Models & Mechanisms • Supplementary information

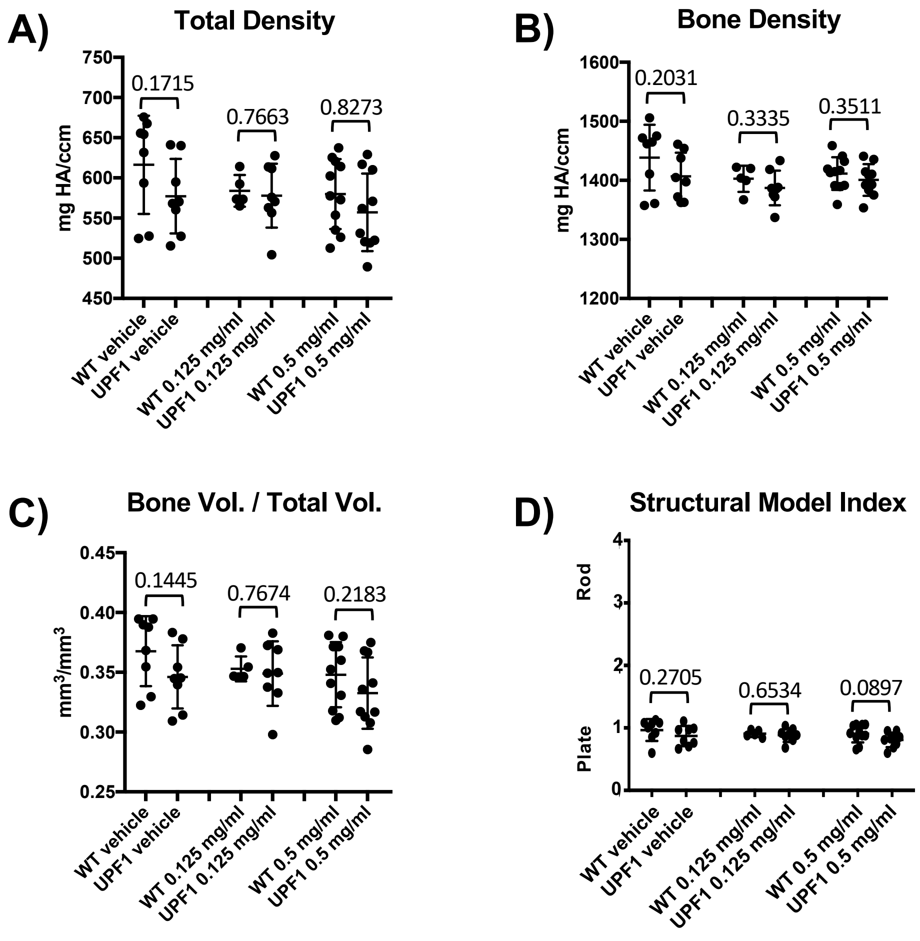

Fig. S9: *Micro-computed tomography of cortical bone in the female femur.* WT and dnUPF1 mice were treated with vehicle or doxycycline for 28 days prior to analysis. The values are presented as the mean (+/- SD) of the parameter calculated from 5-10 mouse cohort for each experimental condition. Exact p values were determined using an unpaired t-test.

Disease Models & Mechanisms • Supplementary information

Table S1: Histological examination of dnUPF1 mouse tissues.*

Tissue Examined	Histological Results (n=5 mice)
Pancreas	Normal
Spleen	Normal
Kidneys	Normal
Liver	Normal
Thymus	Normal
Trachea	Normal
Thyroid	Normal
Salivary glands	Normal
Seminal vesicle	Normal
Prostate	Normal
Bladder	Normal
Testis	Normal
Epididymis	Normal
Preputial gland	Normal
Small intestine	Normal
Uterus	Normal
Ovaries	Normal
Large intestine	Normal
Stomach	Normal
Heart	Normal
Lung	Normal
Brain	Normal
Skin	Normal
Skeletal muscle	Normal
Femorotibial joint	Normal
Bone	Normal
Bone marrow	Normal
Ears	Normal
Nasal passages	Normal
Head	Normal

*Upon weaning, dnUPF1 mice were treated with 500 mg/mL doxycycline in a sugar-free, cherry-flavored Kool-Aid™ vehicle for 28 days.
